# Bayesian integrative analysis of epigenomic and transcriptomic data identifies Alzheimer's disease candidate genes and networks

**DOI:** 10.1371/journal.pcbi.1007771

**Published:** 2020-04-07

**Authors:** Hans-Ulrich Klein, Martin Schäfer, David A. Bennett, Holger Schwender, Philip L. De Jager

**Affiliations:** 1 Center for Translational & Computational Neuroimmunology, Department of Neurology, Columbia University Irving Medical Center, New York, New York, United States of America; 2 Taub Institute for Research on Alzheimer's Disease and the Aging Brain, Columbia University Irving Medical Center, New York, New York, United States of America; 3 Mathematical Institute, Heinrich Heine University, Düsseldorf, Germany; 4 Rush Alzheimer’s Disease Center, Rush University Medical Center, Chicago, Illinois, United States of America; Tufts University, UNITED STATES

## Abstract

Biomedical research studies have generated large multi-omic datasets to study complex diseases like Alzheimer’s disease (AD). An important aim of these studies is the identification of candidate genes that demonstrate congruent disease-related alterations across the different data types measured by the study. We developed a new method to detect such candidate genes in large multi-omic case-control studies that measure multiple data types in the same set of samples. The method is based on a gene-centric integrative coefficient quantifying to what degree consistent differences are observed in the different data types. For statistical inference, a Bayesian hierarchical model is used to study the distribution of the integrative coefficient. The model employs a conditional autoregressive prior to integrate a functional gene network and to share information between genes known to be functionally related. We applied the method to an AD dataset consisting of histone acetylation, DNA methylation, and RNA transcription data from human cortical tissue samples of 233 subjects, and we detected 816 genes with consistent differences between persons with AD and controls. The findings were validated in protein data and in RNA transcription data from two independent AD studies. Finally, we found three subnetworks of jointly dysregulated genes within the functional gene network which capture three distinct biological processes: *myeloid cell differentiation*, *protein phosphorylation* and *synaptic signaling*. Further investigation of the myeloid network indicated an upregulation of this network in early stages of AD prior to accumulation of hyperphosphorylated tau and suggested that increased *CSF1* transcription in astrocytes may contribute to microglial activation in AD. Thus, we developed a method that integrates multiple data types and external knowledge of gene function to detect candidate genes, applied the method to an AD dataset, and identified several disease-related genes and processes demonstrating the usefulness of the integrative approach.

## Author summary

Recent technological advances have led to a new generation of studies that interrogate multiple molecular levels in the same target tissue of a set of subjects, generating complex multi-omic datasets with which to study disease mechanism. These datasets of genetic, epigenomic, transcriptomic, and other data have the potential to reveal novel biological insights; however, integrative analyses remain challenging and require new computational methods. We developed an integrative Bayesian approach to detect genes with consistent differences between case and control samples across multiple data types. The method further integrates prior knowledge about gene function in the form of a gene functional similarity network to improve statistical inference by sharing information between related genes. We applied our method to an Alzheimer’s disease dataset of epigenomic and transcriptomic data and detected and then validated several novel and known candidate genes as well as three major disease-related biological processes. One of these processes reflected microglial activation and included the cytokine *CSF1*. Single-nucleus data revealed that *CSF1* was primarily upregulated in astrocytes, implicating the involvement of this cell type in microglial activation. Hence, we demonstrated that integrative analysis approaches to multi-omic datasets can improve candidate gene detection and thereby generate new insights into complex diseases.

## Introduction

Alzheimer’s disease (AD) is a complex progressive neurodegenerative disease characterized clinically by impaired episodic memory and other impaired cognitive abilities [1]. To better understand disease mechanisms and to identify novel therapeutic targets, several studies of aging and AD have generated large molecular datasets from blood and/or post-mortem human brain samples. Some of these studies targeted multiple molecular levels and measured, for example, genetic variants, epigenetic modifications, mRNAs, or proteins, in the same set of samples [2–4]. However, jointly analyzing genome-wide multi-omic datasets remains challenging and requires novel computational methods to fully utilize these datasets [5].

Integration of multiple data types from the same set of samples has been referred to as vertical data integration [6]. Methods for vertical data integration can be further characterized by the primary goal of the integrative analysis as outlined in recent reviews [5–11]. While the main objective of the method presented in this work is the detection of genes with consistent differences between cases and controls in multiple data types, we review a wider range of vertical data integration methods in the introduction with a focus on those that were either successfully applied to data from AD or related complex diseases or share methodological similarity with our approach. Among the most frequently used are methods for integrating genetic and transcriptomic data. These have successfully identified genetic variants that affect gene transcription thereby improving our understanding of how the transcriptome mediates the effect of risk variants for various diseases including AD [12–14]. Most of these methods regress gene transcription on one or more genetic variants. A simple approach for integrating epigenomic and transcriptomic data is to replace the genetic with epigenetic measurements in the regression model [15–17]. Stepwise regression procedures were proposed to study interaction effects between different histone modifications that may not act independently on gene transcription [18]. Moreover, various machine learning approaches were applied to predict gene transcription levels based on transcription factor binding, histone modification or other epigenomic data [19, 20]. If the primary goal is to predict the effect of an intervention experiment, the often unknown causal structure between different variables has to be learned [21]. Recently, Bayesian networks were applied to infer directed gene-wise graphs that model the relationships between epigenomic, transcriptomic, pathologic and clinical variables in the AD brain [22]. In the classic gene prioritization setting, the primary goal of an integrative analysis is to detect genes with consistent differences between case and control samples across different data types. The motivation for integrating data in this setting is twofold. First, gene prioritization can be improved assuming that a gene with differences in more than one data type is more likely to be a true positive finding. Second, if a specific epigenetic mechanism of a drug is known, target genes for this drug should ideally not only demonstrate differences in the epigenetic data, but also consistent differences at a functionally more relevant level such as gene transcription or protein expression [23]. Methods for detecting differential genes in a joint analysis of multiple data types were developed for experiments with only a few or no replicates [24, 25]. These methods provide probabilistic frameworks for studying the relationship between data types and for classifying genes, but since heterogeneity among replicates is not modelled, statistical inference is challenging and can only be carried out by relying heavily on prior information.

Further need for data integration stems from our constantly increasing knowledge about gene functions and pathways which can be represented as a network where functionally related genes are connected by edges [26]. This prior knowledge can be integrated into an analysis to share information between related genes and thus improve statistical inference and interpretability of the results. For example, gene networks have been used to improve parameter estimation and gene selection in penalized regression models [27–29]. In Bayesian models, conditionally autoregressive (CAR) Markov random field priors were frequently used to incorporate gene networks into genome-wide data analyses [25, 30–32].

Here, we propose a new integrative method to detect genes with consistent differences between case and control groups across multiple data types. The method is based on an integrative coefficient that summarizes information from multiple data types in a case-control study design. To improve statistical inference, a CAR prior is used in a hierarchical Bayesian model to share information between functionally similar genes defined by a gene network. We applied our method to a large AD case-control study consisting of histone ChIP-seq, DNA methylation, and RNA-seq profiles from 233 subjects. Identified genes were validated using protein data and two independent RNA-seq studies of AD. Finally, in a post hoc analysis, we identified differential networks reflecting AD-related processes. Our new method is outlined in the flow chart S1 Fig and described in detail in the Methods section. The validation of our method and findings from the analysis of the AD data are presented in the Results section. We note that our approach can be adapted and applied to other similar multi-omic case-control studies.

## Methods

### Coefficient for integration of multiple genomic variates

We propose an integrative coefficient *Z* that summarizes observations made in different genomic data types for the same subjects and genes in a case-control study. Let $X_{ij}^{\left(k\right)}$ denote the value observed for gene *i* in individual *j* of the patient group in data type *k*, and $Y_{ij}^{\left(k\right)}$ denotes the respective value in the matched control subject. We define *Z*_*ij*_ as the sum of standardized differences

$$Z_{ij}=\sum_{k=1}^{K}S^{\left(k\right)}\frac{X_{ij}^{\left(k\right)}-Y_{ij}^{\left(k\right)}}{\sigma_{X^{\left(k\right)}Y^{\left(k\right)}}},\;i=1,\ldots ,n;\;j=1,\ldots ,m.$$ (1)

The variances of the differences $\sigma_{X^{\left(k\right)}Y^{\left(k\right)}}^{2}=\frac{1}{nm}\sum_{i=1,\ldots ,n;\;j=1,\ldots ,m}{\left(X_{ij}^{\left(k\right)}-Y_{ij}^{\left(k\right)}\right)}^{2}$ are calculated across all genes and used to standardize the differences which may have a different range of values depending on the data type and technical platform used to generate the data. Whether a positive or negative association is expected between the genomic data types is modelled by the factor *S*^(*k*)^∈{−1,1}.

If the differences $X_{ij}^{\left(k\right)}-Y_{ij}^{\left(k\right)}$ for a gene *i* and individual *j* show consistent directions as modelled by *S*^(*k*)^ across all *K* data types, the absolute value of *Z*_*ij*_ is large. In contrast, a difference in one data type might be cancelled out by a difference in another data type if the directions of the differences do not meet the assumption defined by *S*^(*k*)^. *Z*_*ij*_ is also expected to be close to zero if a gene does not have any differences in any data type. Previous work suggested multiplicative instead of additive coefficients for data integration [24, 25, 33], however, replacing the sum by a product in Eq (1) is a very conservative approach when multiple different data types are modelled. A small difference in a single data type would result in a small multiplicative coefficient even if distinct differences are observed in the other data types. In this work, we jointly analyzed gene transcription, histone modification and CG methylation both at promoters and at exons resulting in *K = 4* different data types. To model the negative association between promoter methylation and transcription [34], we set *S*^(*k*)^ = −1 for promoter methylation and *S*^(*k*)^ = 1 for the other three data types.

### Bayesian hierarchical model

#### Model

The *Z*_*ij*_ are assumed to be normally distributed. The normal distributions' means are regressed on two gene-specific effects, *H*_*i*_ and *U*_*i*_. The former is simply assigned a normal distribution, while the latter represents a spatial effect sharing information between functionally similar genes. Similarity information is extracted from an external gene network where two functionally related genes *i* and *g* are connected by an edge and the weight *ω*_*ig*_ of the edge represents the strength or confidence of the relation,

$$Z_{ij}∼N(\mu_{i},\sigma_{i}^{2}),$$

$$\mu_{i}=\beta_{0}+U_{i}+H_{i},$$ (2)

$$1/\sigma_{i}^{2}∼Gamma(a_{\sigma },b_{\sigma }),$$ (3)

$$U_{i}|U_{r},r\neq i∼N(m_{i},\nu_{i}),$$

$$H_{i}∼N(0,\nu_{H}).$$

The spatially structured effect *U*_*i*_ is given an intrinsic Gaussian CAR prior, and the network's similarity values *ω*_*ig*_ are employed as weights,

$$m_{i}=\frac{\sum_{g\in \delta_{i}}\omega_{ig}u_{g}}{\sum_{g\in \delta_{i}}\omega_{ig}}\;\mathrm{and}\;\nu_{i}=\frac{\tilde{\nu }}{\sum_{g\in \delta_{i}}\omega_{ig}},$$

where *δ*_*i*_ denotes a set of ${\tilde{n}}_{i}$ genes neighboring gene *i* in the gene network. Further, *β*_0_ is assigned an improper flat prior on *R*, and the variances $\tilde{\nu }$ and *ν*_*H*_ are assigned inverse Gamma distributions,

$$\beta_{0}∼R(-\infty ,\infty ),$$

$$1/\tilde{\nu }∼Gamma(a_{\tilde{\nu }},b_{\tilde{\nu }}),$$ (4)

$$1/\nu_{H}∼Gamma(a_{\nu_{H}},b_{\nu_{H}}).$$ (5)

The posterior distributions of the quantities *E*_*i*_≔*U*_*i*_+*H*_*i*_,*i* = 1,…,*n*, are used to classify genes as consistently differential, i.e. as presenting congruent differences between cases and controls across different data types. Specifically, gene *i* is assumed to be consistently differential if the 99% credible interval for *E*_*i*_ lies either above or below zero. An implementation of the model in the BUGS language is given in S1 File.

#### Prior elicitation

The hyperparameters of the distributions for $1/\sigma_{i}^{2},1/\tilde{\nu }$ and 1/*ν*_*H*_ are important as they regulate the degree of confidence in the gene-level data and, additionally, in the functional similarities between transcripts reported by the gene network. We suggest an empirical Bayesian approach where the prior in the model is chosen based on the empirical variance observed in the data. To obtain hyperparameters for $\tilde{\nu }$ and *ν*_*H*_, we decompose the variability of the gene-wise mean coefficients $Z_{i•}=\frac{1}{m}\sum_{j=1}^{m}Z_{ij}$ into a non-structural part and a structural part explained by the neighborhood relationship. The structural part of the variance is used to derive a prior for $\tilde{\nu }$, and the remaining variance is used to derive the prior for *ν*_*H*_. Specifically, we assume

$$Var(H_{i})=\nu_{H}\approx Var(Z_{i•}-{\tilde{m}}_{i})\;\mathrm{and}\;Var(U_{i}|U_{r},r\neq i)=\tilde{\nu }/\omega_{\delta_{i}}\approx Var({\tilde{m}}_{i}),$$

with ${\tilde{m}}_{i}=\sum_{g\in \delta_{i}}\omega_{ig}Z_{g•}/\sum_{g\in \delta_{i}}\omega_{ig}$ and $\omega_{\delta_{i}}=\sum_{g\in \delta_{i}}\omega_{ig}$. The hyperparameters in Eqs (4) and (5) are calculated using that *E*(*X*) = *α*/*β* and *Var*(*X*) = *α*/*β*^2^ for *X*~*Gamma*(*α*,*β*). To solve the equations, $\omega_{\delta_{i}}$ is replaced by the average number of neighbors and the variance of the priors is set to 10^4^.

The parameters $\sigma_{i}^{2}$, *i* = 1,…,*n*, model the variability of *Z*_*ij*_ within a gene across subjects. To derive the hyperparameters in Eq (3), we assume $\sigma_{i}^{2}\approx {\mathrm{median}}_{i\in \{1,\ldots n\}}\frac{1}{m}\sum_{j=1}^{m}{\left(Z_{ij}-Z_{i•}\right)}^{2}$ and use a prior variance of 10^4^. The hyperparameters obtained for the presented analysis are given in S1 Table.

### Data

#### Study cohort and case/control definition

The dataset was taken from the longitudinal Religious Orders Study and Rush Memory and Aging Project (ROS/MAP) [35]. Participants of these two studies were without known dementia at time of enrollment and underwent annual cognitive and clinical tests. The studies were approved by an Institutional Review Board of Rush University Medical Center. All participants signed an informed consent, an Anatomic Gift Act for brain donation, and a repository consent to allow their data and biospecimens to be shared. More information on the study and resources can be found on our Resource Sharing Hub at www.radc.rush.edu. Post-mortem neuropathologic evaluation was performed to assess AD and other brain pathologies common in aging and dementia. Gray matter was dissected from biopsies of the dorsolateral prefrontal cortex (DLPFC) and used to generate profiles of the histone acetylome, DNA methylome, and transcriptome [3]. For this study, we defined AD cases based on the NIA Reagan diagnosis (*high* or *intermediate* likelihood of AD) [36] and on the clinical diagnosis of dementia status at time of death (*AD and no other cause of cognitive impairment*) [37]. Subjects with a NIA Reagan diagnosis of *low* likelihood of AD or *no AD* and a clinical diagnosis of *no cognitive impairment* were considered as controls (persons with mild cognitive impairment were excluded from these analyses). This definition of AD and control cases resulted in a total of 141 AD cases (40 males, Ø 90.1 years; 101 females, Ø 91.7 years) and 92 control cases (34 males, Ø 83.0 years; 58 females, Ø 85.9 years) who had complete genome-wide molecular data after quality control. For matching AD cases to controls, age of death was stratified into five-year intervals between 70 and 95 years and an additional >95 years stratum. Then, each AD case was randomly matched to a control of the same gender and age stratum.

#### Matching of data types

**Fig 1:**
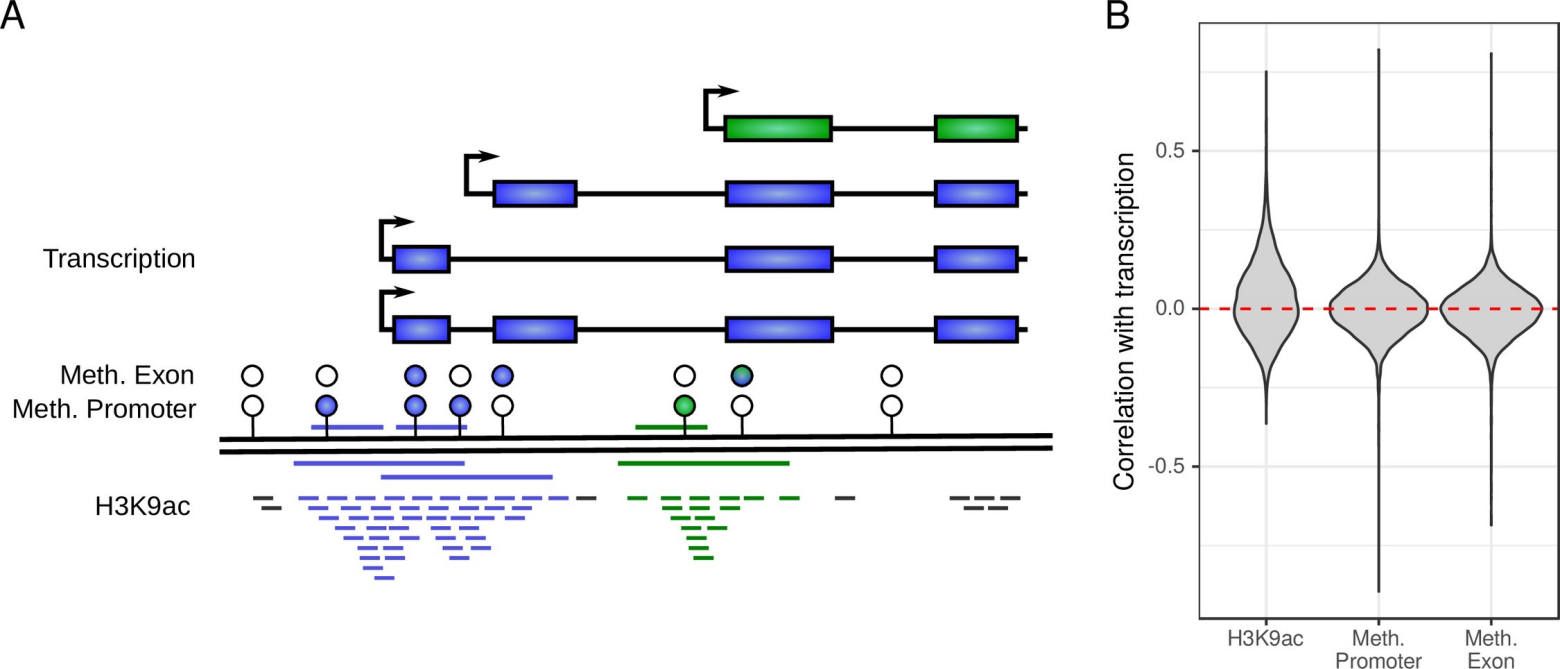
Matching different data types to genes. (A) The figure shows an exemplary gene with four transcripts and their TSSs (small arrows), CG methylation probes (circles), and H3K9ac ChIP-seq reads (small dashes at the bottom) aligned to the genome (black double line). H3K9ac data is matched to transcripts by counting the number of reads in the promoter region (long blue and green lines below the genome). Since the promoter regions (±2.5 kbp around TSS) of the three blue transcripts overlap, the blue transcripts are merged and all ChIP-seq reads are added together. Transcipt-level expression values from RNA-seq data for the blue transcripts are summed accordingly, whereas the green transcript constitutes a separate feature in the final dataset. Methylation levels are calculated separately for promoter and exon methylation. Promoter methylation is calculated as the average methylation level of all probes in the 2 kbp upstream promoter regions of the transcripts (blue and green lines above the genome). Selected probes are indicated by blue and green circles (lower row). Similarly, exon methylation is calculated as the average methylation level of all probes in the respective transcripts’ exons (blue and green circles in the upper row). (B) Violin plots show the correlation between transcription data and H3K9ac, promoter methylation, and exon methylation respectively. Pearson correlation was calculated for each gene across the n = 233 subjects after removing the effects of technical variables, proportion of neurons, age and gender.

Different genomic data types were matched at the transcript (isoform) level following a previously suggested approach [38]. Transcript abundances were estimated from RNA-seq data using RSEM [39]. Only active transcripts with an fpkm value ≥2 in at least 25% of the samples were considered. The targeted histone mark histone 3 lysine 9 acetylation (H3K9ac) is primarily located at active transcriptional start sites (TSS) [40]. To quantify the H3K9ac level for a given transcript *i*, we counted the number of ChIP-seq reads aligned within a genomic region $R_{i}^{H}$ of 5,000 bp centered at the transcript's TSS. Often, multiple transcripts of a gene share the same TSS or have TSSs in close genomic vicinity. These transcripts cannot be distinguished at the H3K9ac level, and thus, we merged genomic regions $R_{i}^{H}$ of two or more transcripts if they overlapped and summed their respective fpkm values (Fig 1A). In total, we observed 23,674 active transcripts which were merged to 14,796 groups of transcripts with disjoint promoter regions $R_{i}^{H}$, and hence, distinct H3K9ac values. DNA methylation levels were measured by the Illumina HumanMethylation450 BeadChip limiting the methylation data to CG dinucleotides included in the chip design. DNA methylation in promoter regions is generally assumed to be negatively correlated with transcription, whereas the correlation with transcription has been reported to be positive when looking at exon methylation [34]. We calculated the promoter methylation for a transcript by averaging the methylation values of all probes located within the promoter region $R_{i}^{M_{1}}$ defined as 2,000 bp upstream of the transcript's TSS. Similarly, exon methylation was calculated by averaging the methylation values of all probes located within the transcript's exonic region $R_{i}^{M_{2}}$. As illustrated in Fig 1A, for groups of transcripts, we combined the regions $R_{i}^{M_{1}}$ and $R_{i}^{M_{2}}$ of the individual transcripts. Overall, we obtained 10,857 transcripts or groups of transcripts with non-missing values for all four data types. For simplicity, we will use the term gene hereafter ignoring the detail that our features actually represent either a single transcript or a group of transcripts that share a promoter, and thus, that a gene with two or more active promoters is represented by two or more features in our dataset.

#### Data normalization

Large genomic datasets are inevitably affected by technical confounders. To reduce the effect of these covariates, we used the preprocessed datasets after quality control as described in the respective original publications [41–43] and subsequently regressed out technical covariates, biological covariates, and the estimated proportion of neurons in the cell type composition of the neocortical tissue. Pearson residuals obtained from the regression models were then plugged into Eq (1) as our normalized observations $X_{ij}^{\left(k\right)}$ and $Y_{ij}^{\left(k\right)}$. Fig 1B depicts the correlation of the residuals of the same gene between different data types. Correlation between the residuals from gene transcription and H3K9ac data were shifted towards positive values, whereas correlation coefficients were almost centered for promoter and exon methylation. A strong correlation is not expected for the majority of genes, since we regressed out known factors like gender that impose a correlation structure between the data types. The remaining correlation structure is caused by AD or by unknown environmental and genetic factors. Environmental and genetic factors likely have a small effect due to the homogeneity of the ROS/MAP cohort.

In more detail, the following regression models were used. For the RNA-seq data (SynapseID: syn3388564), we log-transformed the transcript-level fpkm values and fitted a linear regression model for each transcript with the covariates RNA integrity score, log-transformed total number of sequence reads, batch, postmortem interval, age of death, gender and proportion of neurons. For the ChIP-seq data (Synapse ID: syn4896408), we used the number of reads observed in the genomic region $R_{i}^{H}$ as outcome and fitted a negative binomial regression model for each transcript with the log transformed total number of reads as offset and the covariates cross correlation, postmortem interval, age at death, gender and proportion of neurons. DNA methylation data (Synapse ID: syn3157275) contained methylation values between 0 and 1 for each CG dinucleotide. We applied beta regression models with the covariates bisulfite conversion rate, batch, postmortem interval, age at death, gender and proportion of neurons.

The proportion of neurons used in the regression models to adjust for changes in cell type composition during the course of AD were estimated from the RNA-seq data. We applied the Digital Sorting Algorithm (DSA) [44] to the expression values of the five neuronal marker genes *GABBR2*, *MYT1L*, *ARL4C*, *CADPS* and *NRXN3* that were previously identified using an external human brain RNA-seq reference dataset of purified cells [42, 45]. We observed a decrease of the proportion of neurons from 66.9% to 65.5% in AD subjects (p = 0.01, Wilcoxon rank-sum test, n = 141 AD subjects and 92 controls) indicating the need to adjust for neuronal proportion. RNA-seq-derived estimations were also used to adjust the H3K9ac and DNA methylation data since these data were generated from adjacent specimens of the same tissue block.

### Functional gene network

Information about the functional similarity of genes was obtained from the HumanNet [46]. HumanNet is a functional gene network consisting of 16,243 genes connected by 476,399 weighted edges. Weights *ω*_*ig*_ range between 0.41 and 4.26 (Ø 1.14) and reflect the likelihood of a functional linkage between the two connected genes. The HumanNet was not developed specifically for the human brain and many genes and functional relationships are not observed in the human neocortex. Therefore, we first removed all genes that were not detected in our data. Then, edges of the induced subgraph were removed if the connected genes did not show a correlation coefficient larger or equal to 0.35 (85^th^ percentile) in an external gene transcription dataset from the Mount Sinai Brain Bank (MSBB) AD study [47]. The MSBB dataset consisted of 753 RNA-seq profiles of human aged and Alzheimer’s brain samples generated from four different brain regions (S1 File). The modified network consisted of 6,470 genes connected by 41,093 edges with a mean weight of 1.24. The remaining 4,387 genes in our dataset that were either not represented in the original network or not connected by any edges after pruning remained in the analysis, but for these genes the structural component *U*_*i*_ is ignored in Eq (2).

### Detection of differential subnetworks

We applied the prize-collecting Steiner tree (PCST) algorithm implemented in the R package PCSF [48] to detect subnetworks that were enriched with consistently differential genes identified by our integrative Bayesian analysis. To detect multiple trees in the network, the algorithm introduces an extra root node connected to each node in the network with cost *ω*_0_ [49]. After the PCST problem has been solved, the artificial root node is removed from the tree and the remaining forest structure *F* is returned. Specifically, the algorithm maximized the objective function

$$f(F)=\beta \sum_{i\in V_{F}}\left|{\hat{E}}_{i}\right|-\sum_{(i,j)\in L_{F}}c(\omega_{ij})-\omega_{0}\kappa_{F}$$

where *V*_*F*_ denotes the set of all vertices (genes) in the forest and *L*_*F*_ the set of all edges (functional links between genes) in the forest. Variable *κ*_*F*_ denotes the number of trees in the forest. The cost for an edge *(i*, *j)* was defined as *c*(*ω*_*ij*_) = *ω*_*c*_−*ω*_*ij*_ with constant *ω*_*c*_ set to the sum of the maximum and minimum weight observed in the functional similarity gene network. The optimal solution depends on the two tuning parameters *β* and *ω*_0_ that need to be specified. *β* balances the prizes associated with genes, i.e., the absolute values of the integrative statistics $\left|{\hat{E}}_{i}\right|$, and the costs assigned to the edges. A larger value of *β* results in larger trees. We set *β* = 22, which corresponded to the 75^th^ percentile of the costs *c*(*ω*_*ij*_) divided by the 95^th^ percentile of the prizes $\left|{\hat{E}}_{i}\right|$ in our data. The parameter *ω*_0_ defines the costs for adding a tree to the forest. A larger value of *ω*_0_ results in fewer trees in the optimal solution *F*. We set *ω*_0_ to the 99^th^ percentile of $\beta |{\hat{E}}_{i}|$. Our choices for *β* and *ω*_0_ preferred a solution with a few small trees, which is often better for biological interpretability. The optimal forest *F* with these settings consisted of nine trees. Three trees consisted of ten or more genes and were studied in more detail. These three trees were extended into the subnetworks by adding all edges to a tree that existed between any two genes of the tree in the initial functional gene similarity network. Thus, each of the three subnetworks corresponds to one single tree found by the PCSF algorithm with the identical set of genes but additional edges. Differential subnetworks were tested for an enrichment of gene ontology (GO) terms from the biological process ontology using the R package topGO [50, 51]. Fisher’s test was applied to compare genes within a subnetwork to the background set of all genes included in the initial gene network. GO terms with less than 10 genes were excluded from the analysis.

### Model fitting

The hierarchical Bayesian model was implemented in the BUGS language. The code is available in S1 File. The Gibbs sampler implemented by WinBUGS was used to carry out 400,000 iterations after an initial number of 40,000 burn-in iterations. A thinning of 200 was applied resulting in 2,000 samples from the posterior distributions. Median values and 99% credible intervals were obtained from these 2,000 samples to perform inference. Estimates for the parameters *β*_0_, *ν*_*H*_, and $\tilde{\nu }$, and their respective trace plots are given in S2 Table and S2 Fig.

## Results

### Detection of genes with consistent differences across data types in AD

The primary goal of our integrative analysis was to identify genes with consistent alterations of the epigenome and transcriptome in AD. Evidence for differential gene regulation across transcription, H3K9ac, promoter methylation and exon methylation data was summarized by the integrative coefficient *Z*. In Eq (1), we set *S*^(k)^ = −1 for promoter methylation and *S*^(*k*)^ = 1 for the other three data types to model the negative association between promoter methylation and transcription [34]. Genes were classified as consistently differential if the 99% credible interval for the integrative statistic *E*_*i*_ excluded 0. In total, 393 genes were significantly upregulated and 423 genes were significantly downregulated in AD. S3 Table contains the statistics for all genes included in the analysis. The first ten genes sorted by |*E*_*i*_| are shown in Table 1.

**Table 1:** Top 10 differential genes ranked by $\left|{\hat{E}}_{i}\right|$.

Gene	${\hat{E}}_{i}$ [99% CI]	${\tilde{n}}_{i}$	Ø *ω*_*ig*_	+\-	$Z_{i•}^{\left(\mathrm{exprs}\right)}$	$Z_{i•}^{\left(\mathrm{H3K9ac}\right)}$	$Z_{i•}^{\left(\mathrm{methP}\right)}$	$Z_{i•}^{\left(\mathrm{methE}\right)}$
*SLC6A9*	0.93 [0.49, 1.34]	1	1.05	69.5%	0.72	0.32	-0.07	0.06
*KIF5A*	0.77 [0.43, 1.12]	6	1.41	75.2%	0.69	0.21	-0.28	0.04
*CRB2*	0.76 [0.38, 1.14]	0	-	74.5%	0.53	0.28	-0.17	0.29
*SLC6A12*	0.74 [0.37, 1.13]	0	-	73.8%	0.98	0.33	0.10	0.08
*PRELP*	0.74 [0.40, 1.07]	2	1.13	72.3%	0.67	0.24	-0.07	0.10
*DUSP6*	-0.71 [-1.11, -0.30]	1	0.54	38.3%	-0.18	-0.46	0.09	-0.03
*MAP4K3-DT*	-0.70 [-1.04, -0.36]	0	-	24.8%	-0.56	-0.28	0.07	-0.20
*SLC14A1*	-0.70 [-1.19, -0.16]	1	1.00	28.4%	-1.01	-0.44	0.02	-0.09
*APOD*	0.69 [0.31, 1.06]	4	0.91	67.4%	0.34	0.24	-0.41	0.06
*CDK18*	0.68 [0.32, 1.06]	28	0.78	69.5%	0.68	0.32	-0.06	0.01

Columns from left to right show the gene symbol, estimated integrative statistic ${\hat{E}}_{i}$ and its 99% credible interval, number of neighbors in the gene network, mean weights of neighbors, percentage of subjects *j* with positive coefficient *Z*_*ij*_. The last four columns show the mean coefficient *Z*_*i*•_ calculated using only genes expression, H3K9ac, promoter methylation, and exon methylation data.

Among the differential genes in Table 1 are the neurotransmitter transporters *SLC6A9* and *SLC6A12*, which were associated with cognition in human AD patients and AD model systems [52–54]. A recently developed *SLC6A9* inhibitor is currently tested in a clinical trial [55]. The downregulated phosphatase *DUSP9* and the upregulated kinase *CDK18* have been suggested to modulate pathological tau phosphorylation in AD [56, 57]. *KIF5A* is a motor protein that has been reported to be upregulated in AD and may contribute to AD-related mitochondrial defects [58, 59]. Another candidate gene is *APOD*, which has a neuroprotective role and is upregulated in the aging and AD brain [60, 61]. Overall, the differential genes identified by our analysis are involved in various biological processes in different cell types reflecting the complexity of AD. Before we highlight some of these processes and discuss novel insights, we first study and validate our integrative model in more detail.

The integrative statistic *E*_*i*_ consists of a non-structural component *H*_*i*_ and a structural component *U*_*i*_ defined by the functional similarity gene network. If functionally related genes demonstrate congruent up- or downregulation in AD, we expect to observe large absolute values for *U*_*i*_. To assess the importance of the network in our analysis, we approximated the fraction of the variance of *E*_*i*_ that is contributed by *U*_*i*_, *Var*(*U*_*i*_)/(*Var*(*U*_*i*_)+*Var*(*H*_*i*_)) = 0.06. A fraction of 6% indicates that while *H*_*i*_ accounted for most of the observed differences between AD and controls, some functionally related genes were jointly dysregulated in AD. The structural component *U*_*i*_ alone was significant for a total of 6 genes.

### Assessment of specificity and sensitivity using simulated data

To validate our model and prior development, we simulated a dataset based on the 92 control subjects, which were randomly split into 46 cases and 46 controls. A total of 10,000 genes were randomly selected and assigned to 500 pathways with 20 genes each. Differences between cases and controls were simulated for half of the pathways by adding or subtracting a value Δ to the observed variables $X_{ij}^{\left(k\right)},k=1,\ldots ,4$, of all genes in the pathway in the case samples. Then, a noisy gene network was simulated consisting of 47,500 correct edges between genes of the same pathway and 99,800 incorrect edges between genes of different pathways.

**Fig 2:**
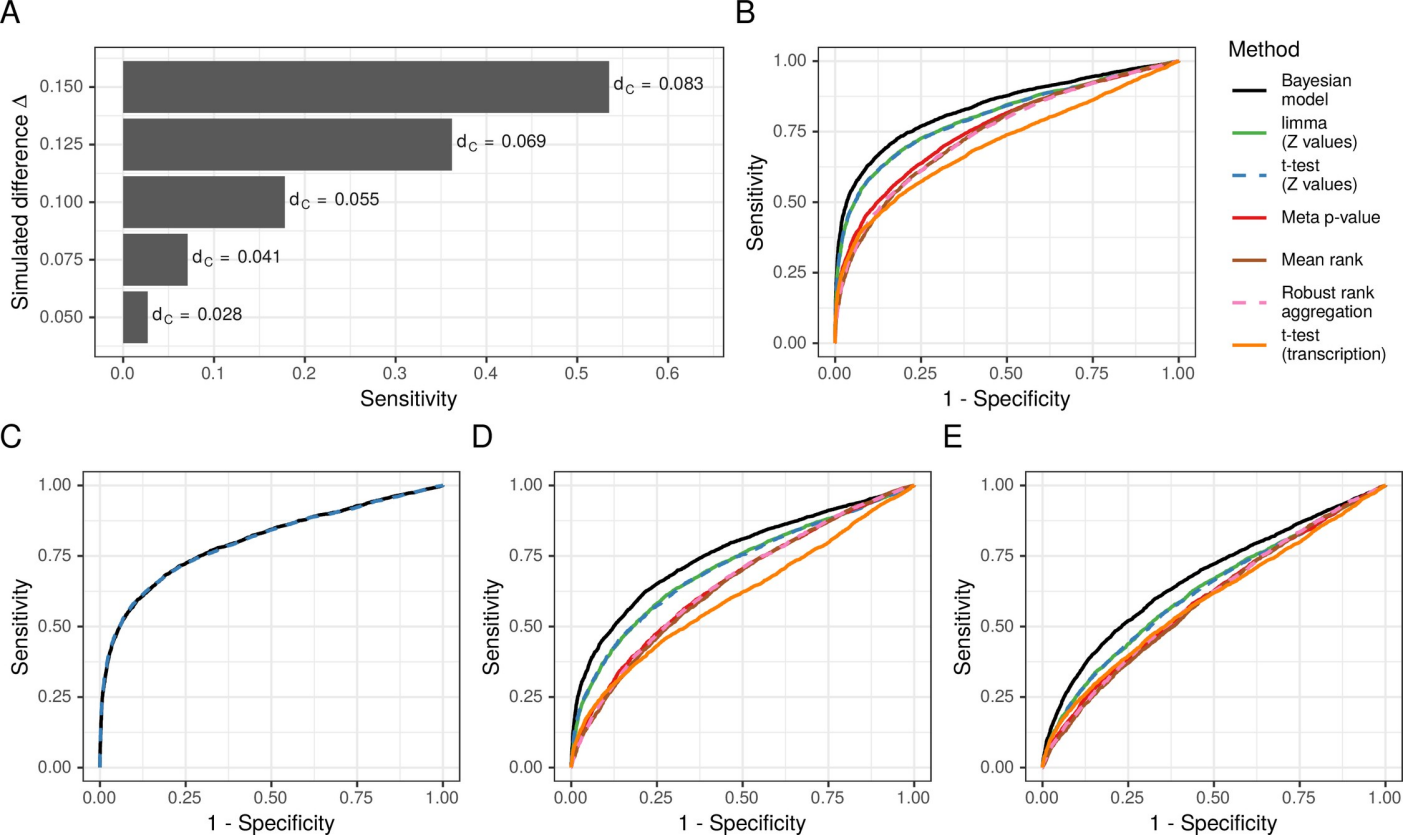
Sensitivity and specificity analysis. (A) The sensitivity achieved by the Bayesian model on the simulated dataset (n = 92) is shown on the x-axis for various simulated differences Δ on the y-axis. The standardized effect size d_C_ (Cohen’s d) is depicted next to the bars. (B) Sensitivity is plotted against 1—specificity as observed in the simulated data for the Bayesian model and six alternative approaches. (C) Sensitivity is plotted against 1—specificity observed when using a random gene network. For better comparison, the curve observed for the t-test identical as in (B) was added to the plot. (D, E) Sensitivity is plotted versus 1—specificity as in (B) using a smaller sample size of n = 46 (D) and n = 20 (E).

Only 19 out of the 5,000 non-differential genes were falsely classified as differential based on a 99% credible interval. The method correctly identified 1,173 of the 5,000 differential genes resulting in an FDR of 0.016 and a sensitivity ranging from 0.027 to 0.535 depending on the magnitude of the simulated difference Δ as depicted in Fig 2A. Notably, even the largest simulated difference Δ = 0.15 corresponded to a small standardized effect size of 0.083 (Cohen’s d). Next, we compared the performance of our Bayesian model to alternative approaches using our simulated dataset. For our Bayesian approach, we observed an area under the receiver operating characteristic curve (AUC) of 0.84 (Fig 2B), which is a modest improvement over the gene-wise one-sample t-tests applied to the *Z* values (AUC of 0.80). A moderated t-test on the *Z* values as implemented in the limma software performed equally well as the regular t-test (AUC of 0.80) [62]. All three approaches operate on the integrative coefficient *Z* and therefore leverage information from all four data types and consider the directionality of the effects observed in the different data types. Alternatively, an integrative analysis can be performed as a meta-analysis on significance levels or rankings obtained from separate analyses of the different data types. To implement this strategy, we first ran paired two-tailed t-tests on the simulated $X_{ij}^{\left(k\right)}$ and $Y_{ij}^{\left(k\right)}$ values separately for each of the *k* = 1,…,4 data types to obtain a p-value per gene and data type. Subsequently, the results from the different data types were combined by either calculating meta-p-values using the z-score method (AUC of 0.76), or by calculating the genes’ mean ranks (AUC of 0.75), or by applying the Robust Rank Aggregation method (AUC of 0.75). The latter method was specifically developed for integrating multi-omic data [63]. However, when combining p-values or gene ranks, information about the directionality of the effect sizes observed in the different datasets is lost resulting in lower AUC values. Finally, we added the results obtained from applying a t-test to only one data type (transcription data as example) to our comparison in Fig 2B. As expected, the AUC of 0.71 was smaller compared to the integrative methods, since a large fraction of the data was not used.

To study how the Bayesian method performs if a non-informative network is given, we randomly permuted the edges of the simulated gene network. Fig 2C shows the results from the Bayesian method with the random network (AUC of 0.80) next to the unchanged results from the t-test for a better comparison. These results indicate that the improvement of the Bayesian method depicted in Fig 2B stems from the information provided by the gene network, and that if a random network is given, the Bayesian methods performs equally well as the t-test. Finally, we studied the effect of different sample sizes on the methods by repeating the simulation study on subsets of n = 46 (Fig 2D) and n = 20 (Fig 2E) samples. While the performance of all methods declined with smaller sample sizes, the Bayesian method maintained an advantage as more relative weight was given to the prior. In summary, the simulation study demonstrated that the Bayesian model with the selected priors results in a small false positive rate, and, if an appropriate network is given, performs better than simple gene-wise t-tests on the integrative coefficients or methods that integrate p-values or ranks.

### Validation in independent datasets

**Fig 3:**
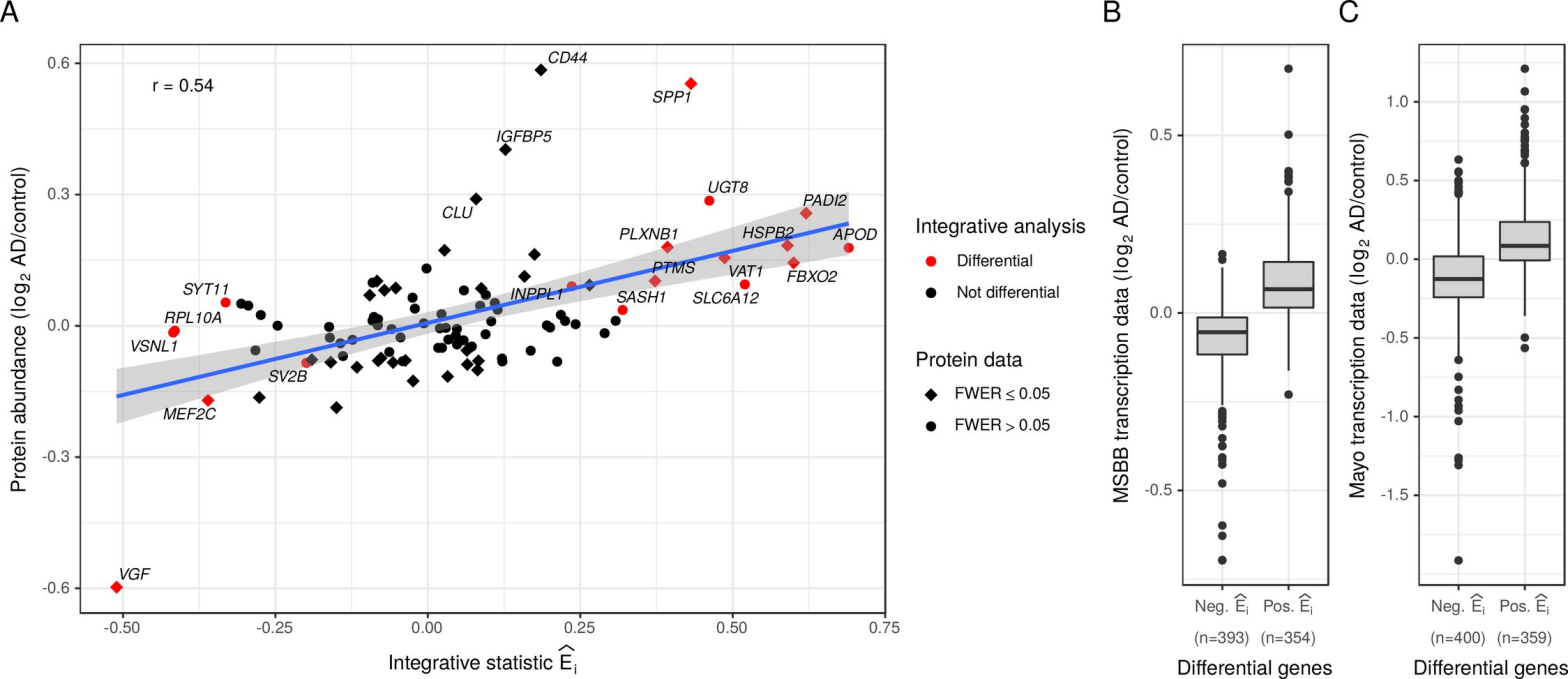
Validation of differential genes identified by the integrative analysis. (A) The integrative statistic for 98 genes that were included in a targeted proteomic dataset is plotted on the x-axis versus the observed differences between AD and control cases in the protein data on the y-axis. Red color indicates genes that were detected as differential in the integrative analysis (n = 233 samples). Squares indicate significant differences in the protein data (n = 607 samples) at a family-wise error rate of 0.05. (B) Differences in gene transcription between AD and controls observed in the MSBB RNA-seq study (inferior frontal gyrus, n = 116 samples) are shown separately for genes identified as up- or downregulated in the integrative analysis. (C) Similarly, differences in gene transcription between AD and controls observed in the Mayo LOAD RNA-seq study (temporal cortex, n = 151 samples) are shown separately for genes identified as up- or downregulated.

We first studied whether genes with consistent differences in epigenomic and transcriptomic data identified by our analysis also presented differences at the protein level. To do this, we utilized a targeted proteomics dataset generated from the same sample type and sample collection as our multi-omic data: DLPFC samples of ROS/MAP participants (Synapse ID: syn10468856). The targeted proteins were candidate genes from previous AD studies [64] and measured by liquid chromatography-selected reaction monitoring [65]. We applied the same case/control definition as for the main analysis resulting in 393 AD cases and 214 control subjects. Each protein was tested for difference in abundance in AD versus control subjects, adjusting for gender, age and postmortem interval (S1 File). Overall, we observed a positive correlation (Pearson’s r = 0.54) between the integrative statistic ${\hat{E}}_{i}$ and the observed differences in the protein data based on 98 proteins encoded by genes considered in our integrative analysis (Fig 3A). Out of 18 differential genes from our integrative analysis, 9 genes demonstrated significantly altered protein levels in AD (family-wise error rate ≤ 0.05); the direction of effect was consistent between the two sets of results.

Since the protein data is limited to selected AD candidate genes and was not generated from an independent cohort, we additionally validated our findings in two RNA-seq datasets from other AD sample collections. The first dataset consisted of 79 AD samples and 37 control samples from the inferior frontal gyrus included in the MSBB study [47]. We identified 747 genes that were classified as differential in our integrative analysis and passed the detection threshold in the inferior frontal gyrus samples of the MSBB dataset (S1 File). When comparing AD to control samples, a majority of 601 out of the 747 genes showed a change in transcription consistent with the results from the integrative analysis (Fig 3B). These changes were significant at an unadjusted p-value of 0.05 for 102 out of 354 upregulated genes and for 97 out of the 393 downregulated genes. Similar results were observed for the second dataset of temporal cortex samples from the Mayo LOAD study (n = 71 control samples, n = 80 AD samples) [4]. We detected 759 of our differential genes in the temporal cortex (S1 File), and 553 of these genes showed a consistent increase or decrease in AD (Fig 3C). At an unadjusted p-value of 0.05, 154 out of 359 upregulated and 200 out of 400 downregulated genes were validated in the Mayo LOAD study.

### Differential subnetworks

When we analyzed the posterior distributions we found that about 6% of the variance of *E*_*i*_ is contributed by *U*_*i*_, suggesting that some parts of the gene network are collectively dysregulated in AD. Such a subnetwork of jointly differential genes often represents a disease-related biological process and is easier to interpret than single genes. To identify differential subnetworks post hoc, we applied a prize-collecting Steiner tree (PCST) algorithm [48, 66]. The objective of the PCST algorithm was to find a subnetwork that maximizes the sum of $\left|{\hat{E}}_{i}\right|$ of the genes in the subnetwork minus the costs for the edges *c*(*ω*_*ij*_) needed to construct the subnetwork.

**Fig 4:**
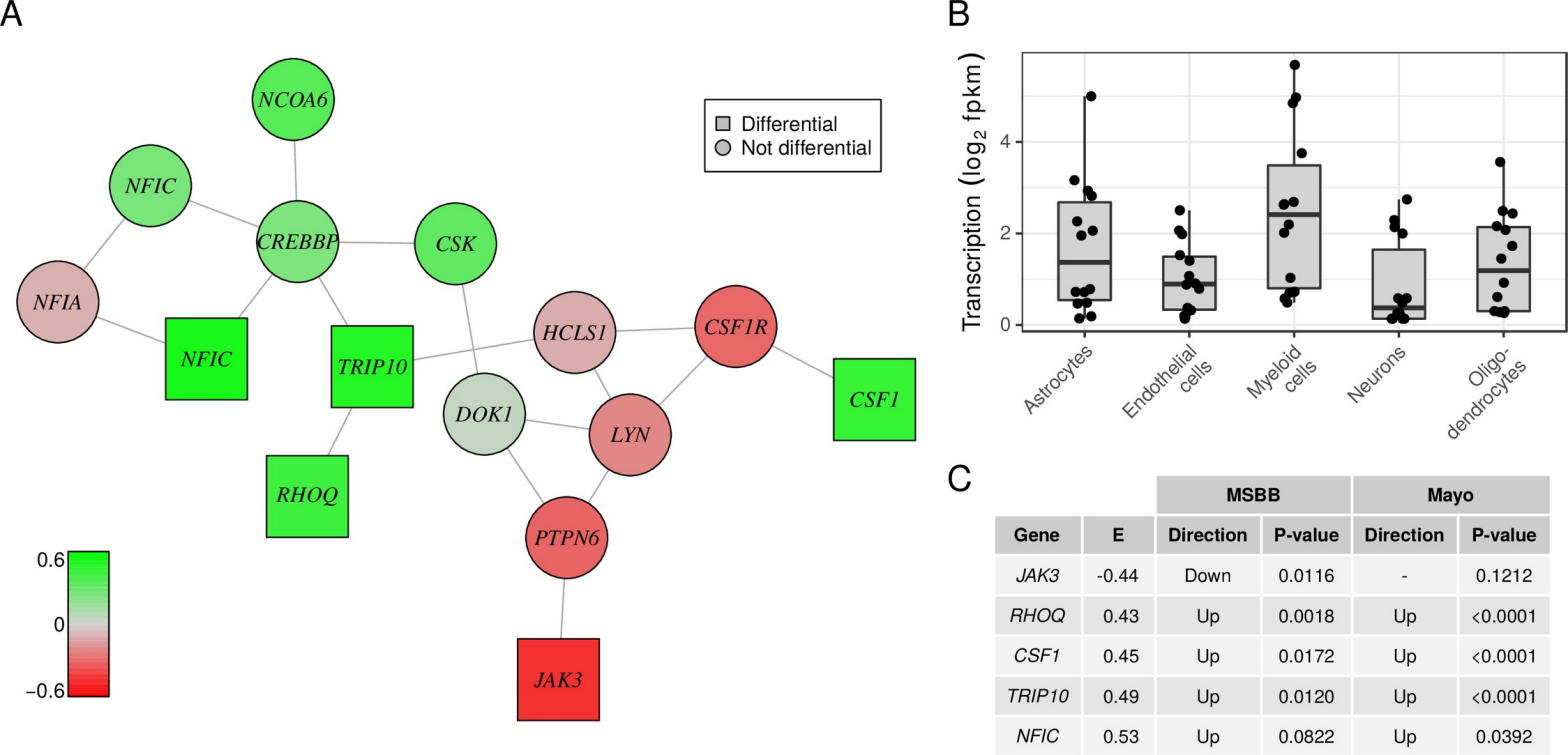
Myeloid cell differentiation network. (A) Graph shows the subnetwork of differential genes largely involved in myeloid cell differentiation. Color encodes the value of the integrative statistic from green (upregulated in AD) to red (downregulated in AD). Squares indicate significantly differential genes (99% credible interval). The gene *NFIC* is represented twice reflecting two alternative active promoters. (B) Boxplots depict the transcription levels of the subnetwork’s genes in each of five major brain cell types obtained from an external RNA-seq dataset of purified cell types. (C) Table shows the value of the integrative statistic ${\hat{E}}_{i}$ and the unadjusted p-value from the two external validation datasets for each significant gene in the subnetwork. The directionality in the validation studies (up- or downregulated in AD) is given if the p-value was less than 0.1.

Three differential subnetworks with at least 10 genes were identified. The first subnetwork (Fig 4A) was enriched with genes involved in *myeloid cell differentiation* (S4 Table) and reflected the immune component of AD [67]. The network included myeloid transcription factors such as *NFIC* [68], and cytokines such as *CSF1* and the corresponding receptor *CSF1R*, which have recently been studied in the context of microglia activation [69–71]. Cellular functions of the upregulated genes *RHOQ* and *TRIP10* include endocytosis and regulation of cell shape and motility [72, 73]. To verify that this gene network is transcribed by myeloid cells, we compared the genes’ transcription levels in an external RNA-seq dataset of purified human brain cells (Fig 4B) [45]. Further, all five significant genes in the network were also differentially transcribed in the MSBB or the Mayo LOAD dataset (Fig 4C). We note that the myeloid genes that we prioritize are different from the well-validated myeloid AD susceptibility genes that have emerged from genome-wide association studies.

The second differential network (S3 Fig) was enriched for the Gene Ontology term *protein phosphorylation* (S5 Table). Protein phosphorylation regulates various cellular processes by altering protein activity, localization and stability, and this mechanism has been implicated in AD [74]. For example, the gene *PRKAA2* (alias *AMPK*) encodes a kinase that regulates cellular energy homeostasis, is activated by amyloid-β, and phosphorylates tau at multiple sites [75–77]. Another kinase directly involved in the phosphorylation and accumulation of tau is TTBK1 [78, 79]. Interestingly, TTBK1 also phosphorylates TDP-43, a protein which forms pathologic aggregates in aged and AD brains [80, 81]. MAP2K4 (alias MKK4) has been suggested to phosphorylate tau [82] and to modulate amyloid-β toxicity [83]. Most kinases and phosphatases were depicted in the right half of the network (S3 Fig). The lower left part of the network included two genes, *TUBA1B* and *TUBB2A*, that encode major constituents of microtubules, which are disrupted by hyperphosphorylated tau in AD [84]. The tubulin genes were connected to the mitochondrial fission gene *DNM1L* (alias *DRP1*) in the network. The protein DNM1L interacts with amyloid-β and hyperphosphorylated tau, causing mitochondria fragmentation and thereby affecting mitochondrial health and axonal transport in AD neurons [85–87]. Thus, the lower left part of the network reflected impaired energy metabolism in AD synapses. The upper left part of the network consisted of dysregulated genes of the ubiquitin proteasomal system, such as *PSMD2*, *BTRC*, *CUL9* and *UBQLN1* [88]. UBQLN1 is involved in the degradation of PSEN1 and APP, two proteins which are essential for the generation of amyloid-β peptides (*APP* is the gene that encodes the amyloid-β peptide) [89, 90]. Overexpression of *UBQLN1* alleviates symptoms in some AD mouse models [91]. Thus, while this complex network captures several different processes, we refer to the network as protein phosphorylation network because of the enrichment with kinases and phosphatases (S5 Table). Many genes of this network were transcribed by neurons (S3B Fig).

The third differential network was characterized by the GO term *synaptic signaling* (S6 Table) and mainly consisted of downregulated synaptic genes (S4 Fig). For example, *RGS7* regulates synaptic plasticity by modulating the signaling pathway downstream of the GABA_B_ receptor [92]. *RPH3A*, another downregulated gene involved in synaptic signaling, correlates with cognitive decline and is specifically downregulated by amyloid-β [93]. Similarly, the glutamate receptors GRIA2 (alias GluR2) and GRIN2A (alias GluN2A) have been shown to be reduced in the postsynaptic density in AD and are associated with memory deficit [94, 95]. Overall, this network, which is mainly transcribed by neurons (S4B Fig), reflects abnormalities in synaptic signaling and a reduction of synaptic density, which is a hallmark of AD and occurs before neuronal cell death [96, 97].

### Upregulation of the myeloid network is associated with amyloid-β pathology and promoted by *CSF1* expressing astrocytes

**Fig 5:**
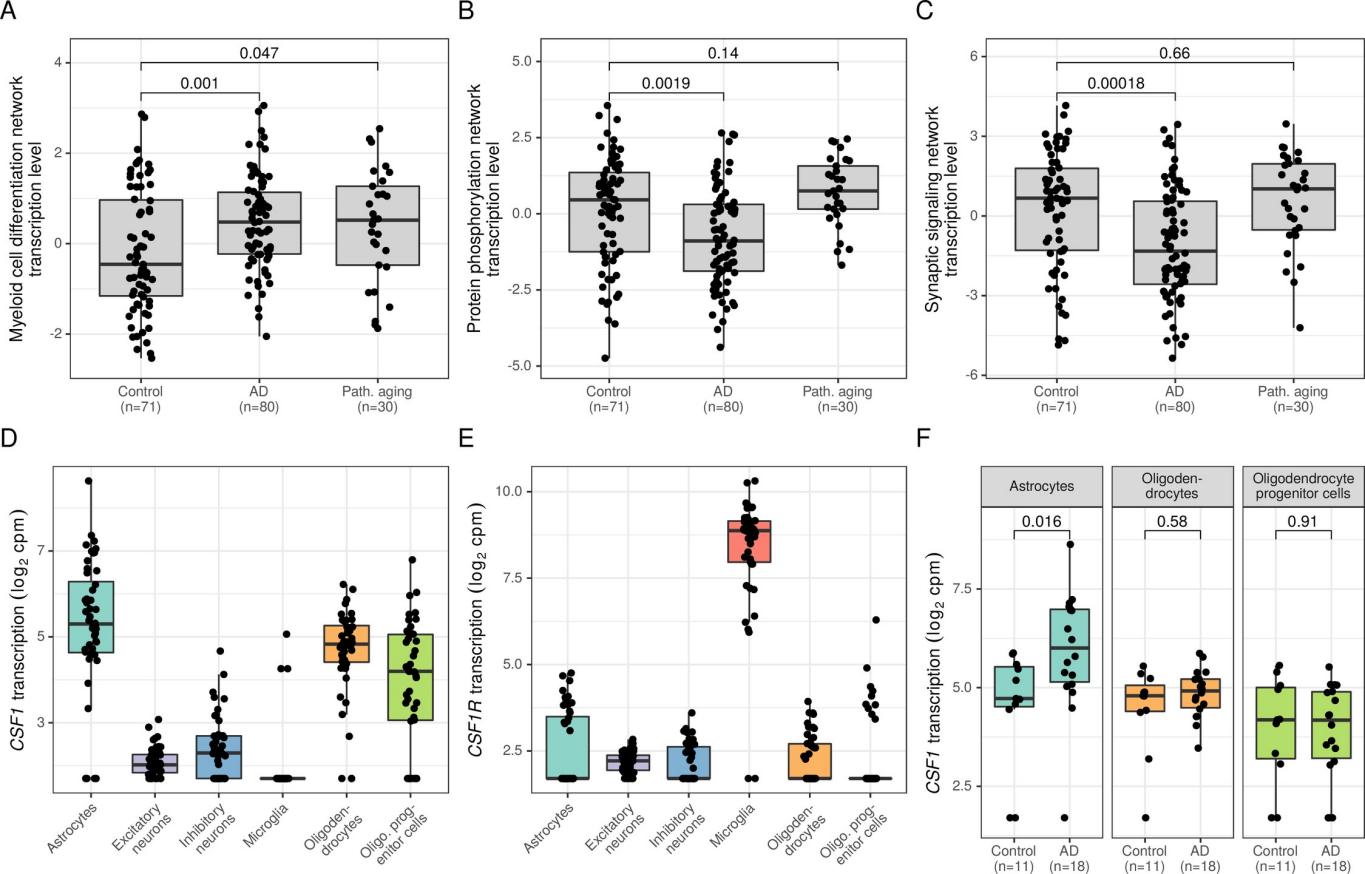
Increased *CSF1* transcription in astrocytes contributes to amyloid-β-related activation of the myeloid cell differentiation network. (A) Boxplots show transcription levels of the myeloid cell differentiation network (first principal component) in control, AD, and pathological aging samples from the Mayo LOAD study (Wilcoxon rank-sum tests, unadjusted p-values). (B, C) Similarly, network transcription levels are shown for the protein phosphorylation network (B), and for the synaptic signaling network (C). (D, E) Boxplots depict transcription levels of *CSF1* (D) and *CSF1R* (E) in six major human brain cell types measured in the prefrontal cortex from 48 individuals. (F) *CSF1* transcription levels are shown separately for controls and AD cases in astrocytes, oligodendrocytes and oligodendrocyte progenitor cells (Wilcoxon rank-sum tests, unadjusted p-values).

To further investigate the role of the biological processes underlying the three differential networks in neurodegeneration, we leveraged the RNA-seq data from the Mayo LOAD study. In addition to 80 AD and 71 control samples, the dataset from the Mayo LOAD study also included 30 samples with a post mortem diagnosis of pathologic aging [4]. Individuals with pathologic aging have widespread cortical amyloid-β plaque deposits but demonstrate no or only minimal neurofibrillary tau pathology and are cognitively non-impaired [98]. It is unclear whether pathologic aging is an early stage of AD or whether this condition develops in individuals who have protective factors that block processes downstream of amyloid-β pathology [99]. We summarized the transcriptional activity of a network in the Mayo cohort by calculating the first principal component of the normalized RNA-seq transcription profiles of the network’s genes (S1 File). First, we verified that the three networks were differentially transcribed between AD and controls in this independent cohort (Fig 5A–5C). As expected, the myeloid cell differentiation network was significantly upregulated in AD compared to controls (p = 0.001, Wilcoxon rank-sum test), and the protein phosphorylation (p = 0.002, Wilcoxon rank-sum test) and the synaptic signaling (p<0.001, Wilcoxon rank-sum test) networks were significantly downregulated in AD. Interestingly, the individuals diagnosed with pathologic aging demonstrated an upregulation of the myeloid cell differentiation network (p = 0.047, Wilcoxon test) to a level similar to that seen in AD subjects (Fig 5A), whereas the protein phosphorylation and synaptic signaling networks were not dysregulated in this group of samples (Fig 5B and 5C). These findings indicate that the upregulation of the myeloid cell differentiation network does not require tau pathology and is probably an early event in the pathogenesis of AD preceding tau pathology and neuronal dysfunction that manifest as impairment in cognitive function. These results are consistent with a recent study suggesting that microglia interact with amyloid-β pathology to contribute to tau proteinopathy and downstream cognitive decline [100].

The myeloid cell differentiation network consisted of 14 genes of which 5 were classified as differential (Fig 4A). The cytokine *CSF1*, one of the 5 differential genes, is an interesting candidate gene, because of its role as a regulator of myeloid cell frequency and function during homeostasis and inflammation [69, 101]. Previous studies of the corresponding receptor *CSF1R* in AD mouse models showed that blocking *CSF1R* reduced microglia density and attenuated the burden of AD pathology in the animals [70, 102]. To investigate which cell types trigger *CSF1* signaling, we employed single-nucleus RNA-sequencing (snRNA-seq) data from n = 48 subjects from the ROS/MAP study (S1 File) [103]. As shown in Fig 5D, *CSF1* was primarily transcribed in astrocytes, oligodendrocytes and oligodendrocyte progenitor cells. Next, we confirmed that *CSF1R* was exclusively transcribed by myeloid cells in the human prefrontal cortex (Fig 5E). Finally, we tested which of the three cell types that transcribed *CSF1* contributed to the differences between AD and controls observed at the tissue level. Interestingly, an upregulation of *CSF1* in AD was only observed in astrocytes (p = 0.016, Wilcoxon rank-sum test), but not in oligodendrocytes or oligodendrocyte progenitor cells (Fig 5F). Although the sample size of the snRNA-seq data is limited, these findings suggest that astrocytes activate microglia cells via *CSF1* signaling. The alternative *CSF1R* ligand *IL34* was not detected as differential in our integrative analysis (S3 Table).

## Discussion

Large multi-omic datasets are becoming more common in biomedical research and require novel integrative bioinformatics approaches to fully harness their potential. We developed an integrative method to detect genes consistently altered in multiple data types in case-control studies. In addition to integrating information from different data types, our method also utilizes functional gene similarity to share information across genes and thereby improve statistical inference.

Information from different data types is aggregated by the integrative coefficient given in Eq (1) at the gene level. Data was matched to genes based on genome annotation in our AD study, however, other data types, e.g. some enhancer marks, may require a more complex matching strategy as outlined elsewhere [38, 104]. After matching, we observed primarily positive gene-wise correlations between transcription and H3K9ac, whereas no clear trend was observed for promoter or exon methylation (Fig 1B). This may reflect the complex relation between DNA methylation and transcriptional activity in the brain, including the role of hydroxymethylation in neurons [105], but we also note that a large correlation between the residuals of different data types should generally not be expected, since we regressed out the effects of major factors such as age, gender and proportion of neurons that impose a correlation structure on the data. The remaining correlation structure was likely caused by unknown genetic and environmental factors as well as the AD status, which may not affect many genes in all data types. To limit the effect of data types that are not associated with the outcome, we modelled an additive instead of a multiplicative coefficient suggested by previous studies [24, 25]. Further, multiplicative coefficients follow a more complex product distribution and the sign of the coefficient is difficult to interpret if more than two data types are involved. In Eq (1), the factors *S*^(*k*)^ model the relationship between data types so that the sign of the coefficient corresponds to an up- or downregulation, respectively. The factors *S*^(*k*)^ can often be chosen based on prior knowledge derived from studies like Encode or Roadmap Epigenomics [106, 107].

A hierarchical Bayesian model is used to study the distribution of the integrative coefficient. An innovation of the model is the representation of the differences between AD cases and controls by a non-structural component *H*_*i*_ and a structural component *U*_*i*_ which shares information between functionally related genes. Based on the results in a recent comparative review, we selected the HumanNet to define functional similarity [108]. Functional similarity networks are constructed from various datasets from different tissues and organisms and are not brain specific. Thus, we had to customize the network and prune edges which were not supported by an external brain RNA-seq dataset in order to better utilize the information contained in the network for our specific analysis. After pruning the network, we observed that overall approximately 6% of the AD effects were contributed by *U*_*i*_ indicating that parts of the gene network were jointly dysregulated in AD. The fraction of variation attributable to the structural component depends on the gene network and the structure of the studied data and may vary between diseases and tissues. In other domains like spatial epidemiology, a wide range of values has been observed that can be as large as 71% in extreme cases [109]. Future studies will have to show whether a fraction of 6% as observed in this study is a common value for genome-wide molecular data.

We validated our model using simulated and independent data from other studies. The simulation study was important to demonstrate that our prior choices result in a reasonable small FDR of 0.016 when using 99% credible intervals to classify genes. Further, the simulation study showed that our method outperforms a one-sample t-test on the integrative coefficients, if an appropriate network is given. The one-sample t-test on the integrative coefficient resembles a paired t-test as the coefficient is the sum of the differences between the matched samples across data types, and thus, can be expected to be powerful in the setting of a matched case-control study. Consequently, when a random network was given, both methods performed equally well indicating that the advantage of the Bayesian method stemmed from the information provided by the network. Methods that integrate results from separate analyses were inferior as these methods ignore the directionality of the observed differences in the different data types. Further, these methods do not provide a statistical framework for assessing significance and controlling error rates. For example, the z-score approach as included in the comparison will likely result in inflated p-values since the data sets are not independent.

Comparing our results from the integrative analysis with the protein data and the external transcription data revealed that a majority of our findings can be reproduced at the protein level and at the mRNA level in an independent cohort, even though the aim of our method was not to predict differences at the protein or transcription level, but to identify genes with consistent differences across the given data types. In line with the validation results, we found that many of the differential genes given in Table 1 have been studied as candidate genes for AD. Thus, we anticipate that the complete result from the gene-wise analysis (S3 Table) is a useful resource for AD candidate genes. However, we take these results further, prioritizing a subset of genes that may be of greater interest: based on the gene-wise results, we studied which parts of the gene similarity network were collectively dysregulated in AD. Three different dysregulated AD subnetworks were identified: *myeloid cell differentiation*, *protein phosphorylation*, and *synaptic signaling*. Similar network-based approaches have been suggested to reveal disease related pathways which may not become obvious in a gene-wise analyses [110]. In contrast to single genes, a network signature can usually be replicated more robustly in model systems or independent datasets, and thus, can be helpful in follow-up studies to address questions such as the temporal progression of these three processes during the course of AD.

In this study, we further investigated the status of the three differential networks in pathologic aging, which is characterized by high amyloid-β loads similar as in AD but a lack of distinct tau pathology [98]. Consistent with the normal cognition of individuals with pathologic aging, the synaptic signaling network was not altered compared to controls. We also found no evidence for altered transcription of the protein phosphorylation network; however, the myeloid cell differentiation network was upregulated to a similar level as observed in AD. Although it is unclear whether the amyloid-β aggregation in pathologic aging reflects an early stage of AD, these findings support the hypothesis that microglia are already activated at the preclinical stage of AD before accumulation of hyperphosphorylated tau. An interesting member of the myeloid cell differentiation network is *CSF1* because of its role as a regulator of myeloid cell numbers and functions [101]. A few studies of AD focused on the corresponding receptor *CSF1R* as a potential therapeutic target. Microglia cells depend on *CSF1R* signaling [111, 112] and treatment of AD mice with *CSF1R* inhibitors results in reduced microglia activation and improved memory function [70, 113], but little is known about the cells that contribute to *CSF1R* triggering in AD. Using snRNA-seq data, we showed that the upregulation of *CSF1* observed at the tissue level is primarily caused by astrocytes in human AD brains. Altogether, our findings suggest that astrocytes contribute to microglial activation by expressing *CSF1* at an early stage of AD preceding tau accumulation. Whether *CSF1* overexpression by astrocytes is directly provoked by amyloid-β cannot be concluded from our data. Interestingly, activated microglia in return secrete signals that induce reactive astrocytes illustrating the complex relationship between these two cell types during the pathogenesis of AD [114].

In summary, we proposed a novel method for the joint analysis of multiple genome-wide datasets that utilizes external information about functional gene similarity. We applied the method to transcription, histone acetylation and DNA methylation data from a large AD study and discovered multiple well-known and new target genes as well as AD processes. Further study of one of these processes indicated that astrocytes may contribute to microglia activation by *CSF1* expression at early stages of AD. Our approach can be adapted to analyze other multi-omic case-control datasets and thereby promotes integrative analyses to fully utilize these complex datasets.

## Supporting information

S1 FigSchematic overview of the integrative multi-omic analysis.Blue boxes indicate input datasets, red boxes indicate our novel integrative analysis, and green boxes indicate the explorative post hoc analysis of differential subnetworks. The genomic data from our AD case-control study consisted of four different data types, which were matched to genes and summarized by the integrative coefficient *Z*_*ij*_. *Z*_*ij*_ modeled the differences observed across data types for gene *i* when comparing sample *j* to its matched control sample. The distribution of *Z*_*ij*_ was modeled by a hierarchical Bayesian model to identify consistently differential genes. The Bayesian model incorporated a gene network (HumanNet) to share information between functionally related genes. Genes with consistent differences in the epigenomic and transcriptomic data between AD and control samples were the primary result of our integrative analysis (S3 Table). To further analyze and interpret the results, we subsequently employed an explorative network-based approach to detect AD-related subnetworks (green boxes). Therefore, we annotated the genes in the HumanNet with the integrative statistic ${\hat{E}}_{i}$ derived from our Bayesian model and used a prize-collecting Steiner tree (PCST) algorithm to identify subnetworks enriched with consistently differential genes.(TIF)

S2 FigTrace plots of MCMC draws.(A) Trace plot for parameter *β*_0_ after removing the burn-in period. A thinning of 200 was applied. (B) Trace plot for parameter *ν*_*H*_ after removing the burn-in period. A thinning of 200 was applied. (C) Trace plot for parameter $\tilde{\nu }$ after removing the burn-in period. A thinning of 200 was applied.(TIF)

S3 FigProtein phosphorylation network.(A) Graph shows the subnetwork of differential genes largely involved in protein phosphorylation. Color encodes the value of the integrative statistic from green (upregulated in AD) to red (downregulated in AD). Squares indicate significantly differential genes (99% credible interval). (B) Boxplots depict the transcription levels of the subnetwork’s genes in each of five major brain cell types obtained from an external RNA-seq dataset of purified cell types. (C) Table shows the value of the integrative statistic ${\hat{E}}_{i}$ and the unadjusted p-value from the two external validation datasets for each significant gene in the subnetwork. The directionality in the validation studies (up- or downregulated in AD) is given if the p-value was less than 0.1.(TIF)

S4 FigSynaptic signaling network.(A) Graph shows the subnetwork of differential genes largely involved in synaptic signaling. Color encodes the value of the integrative statistic from green (upregulated in AD) to red (downregulated in AD). Squares indicate significantly differential genes (99% credible interval). The gene *ANK2* is represented twice reflecting two alternative active promoters. (B) Boxplots depict the transcription levels of the subnetwork’s genes in each of five major brain cell types obtained from an external RNA-seq dataset of purified cell types. (C) Table shows the value of the integrative statistic ${\hat{E}}_{i}$ and the unadjusted p-value from the two external validation datasets for each significant gene in the subnetwork. The directionality in the validation studies (up- or downregulated in AD) is given if the p-value was less than 0.1.(TIF)

S1 Table Hyperparameters of the hierarchical Bayesian model. (DOCX)

S2 Table Parameter estimates of the hierarchical Bayesian model. (DOCX)

S3 Table Analysis results for all 10,857 genes. (XLSX)

S4 Table GO analysis of the myeloid cell differentiation subnetwork. (DOCX)

S5 Table GO analysis of the protein phosphorylation network. (DOCX)

S6 Table GO analysis of the synaptic signaling network. (DOCX)

S1 File Detailed description of the datasets used in this study including data preprocessing. BUGS code for the hierarchical Bayesian model.
(PDF)
