# Supplementary figures and images for: Bayesian integrative analysis of epigenomic and transcriptomic data identifies Alzheimer's disease candidate genes and networks
Source: PLoS Comput Biol. 2020 Apr 7;16(4):e1007771. doi: 10.1371/journal.pcbi.1007771 (PMC7138305; doi:10.1371/journal.pcbi.1007771)

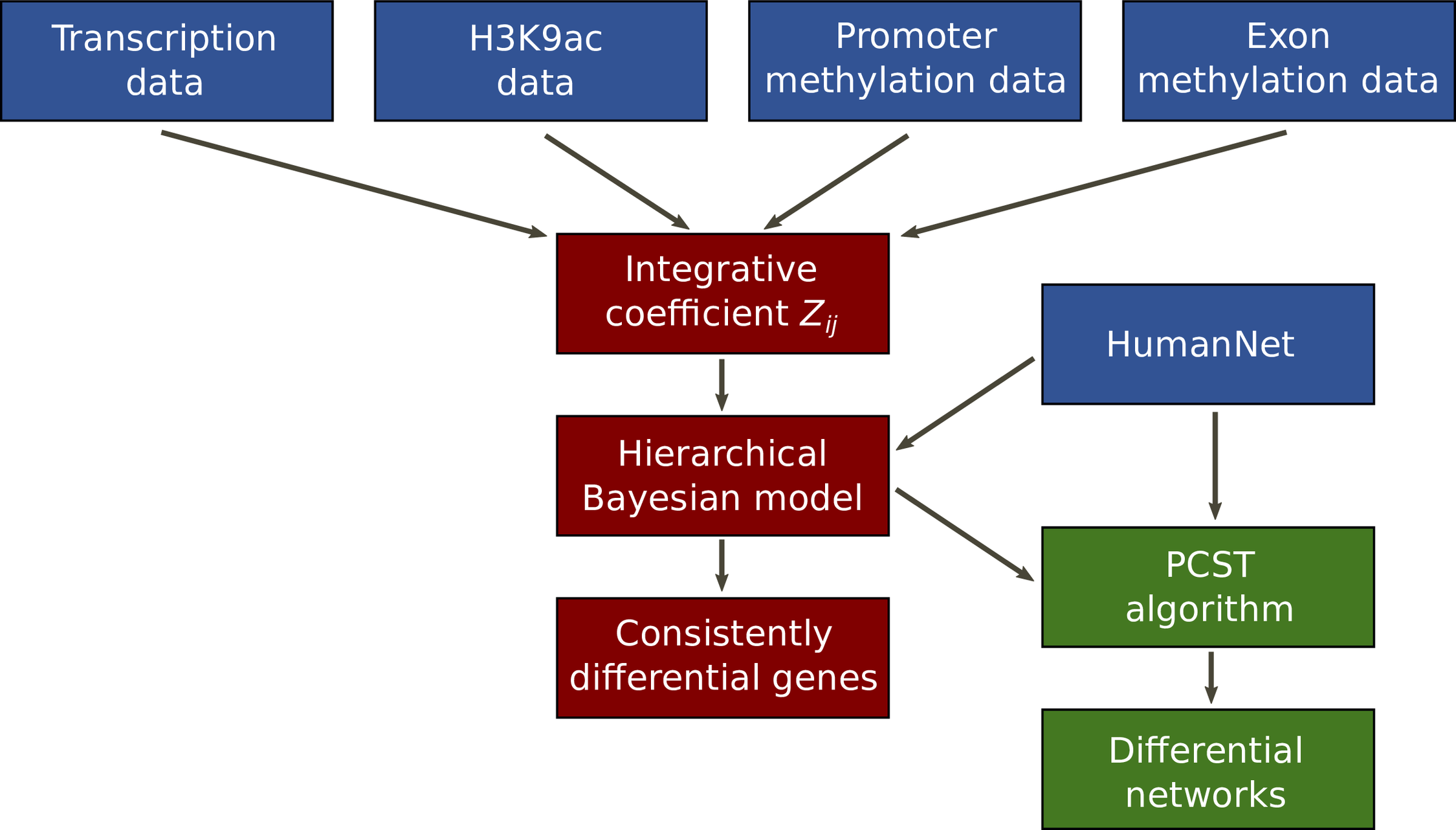

Supplement: S1 Fig — Blue boxes indicate input datasets, red boxes indicate our novel integrative analysis, and green boxes indicate the explorative post hoc analysis of differential subnetworks. The genomic data from our AD case-control study consisted of four different data types, which were matched to genes and summarized by the integrative coefficient Zij. Zij modeled the differences observed across data types for gene i when comparing sample j to its matched control sample. The distribution of Zij was modeled by a hierarchical Bayesian model to identify consistently differential genes. The Bayesian model incorporated a gene network (HumanNet) to share information between functionally related genes. Genes with consistent differences in the epigenomic and transcriptomic data between AD and control samples were the primary result of our integrative analysis (S3 Table). To further analyze and interpret the results, we subsequently employed an explorative network-based approach to detect AD-related subnetworks (green boxes). Therefore, we annotated the genes in the HumanNet with the integrative statistic E^i derived from our Bayesian model and used a prize-collecting Steiner tree (PCST) algorithm to identify subnetworks enriched with consistently differential genes. (TIF) [file pcbi.1007771.s001.tif]

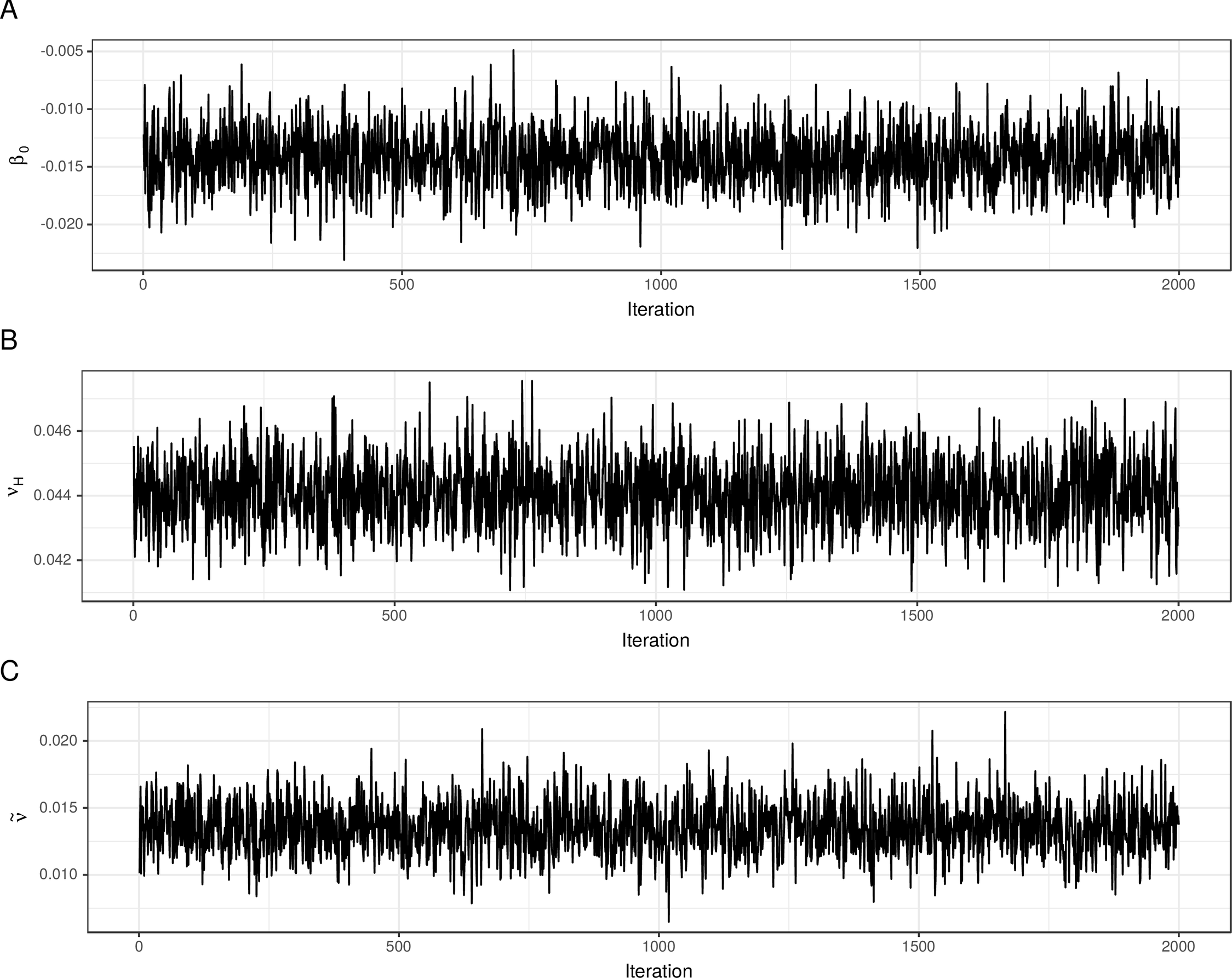

Supplement: S2 Fig — (A) Trace plot for parameter β0 after removing the burn-in period. A thinning of 200 was applied. (B) Trace plot for parameter νH after removing the burn-in period. A thinning of 200 was applied. (C) Trace plot for parameter ν˜ after removing the burn-in period. A thinning of 200 was applied. (TIF) [file pcbi.1007771.s002.tif]

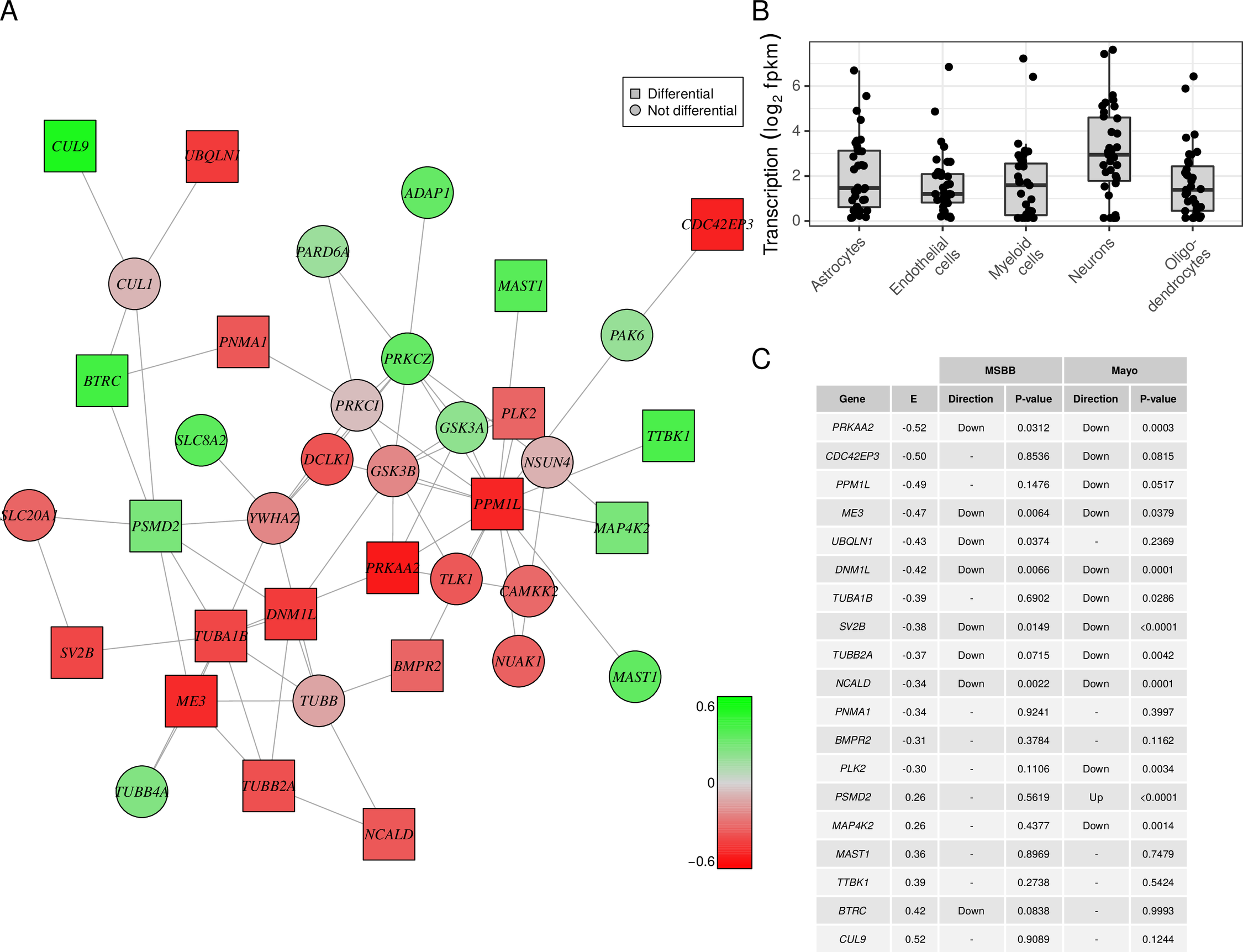

Supplement: S3 Fig — (A) Graph shows the subnetwork of differential genes largely involved in protein phosphorylation. Color encodes the value of the integrative statistic from green (upregulated in AD) to red (downregulated in AD). Squares indicate significantly differential genes (99% credible interval). (B) Boxplots depict the transcription levels of the subnetwork’s genes in each of five major brain cell types obtained from an external RNA-seq dataset of purified cell types. (C) Table shows the value of the integrative statistic E^i and the unadjusted p-value from the two external validation datasets for each significant gene in the subnetwork. The directionality in the validation studies (up- or downregulated in AD) is given if the p-value was less than 0.1. (TIF) [file pcbi.1007771.s003.tif]

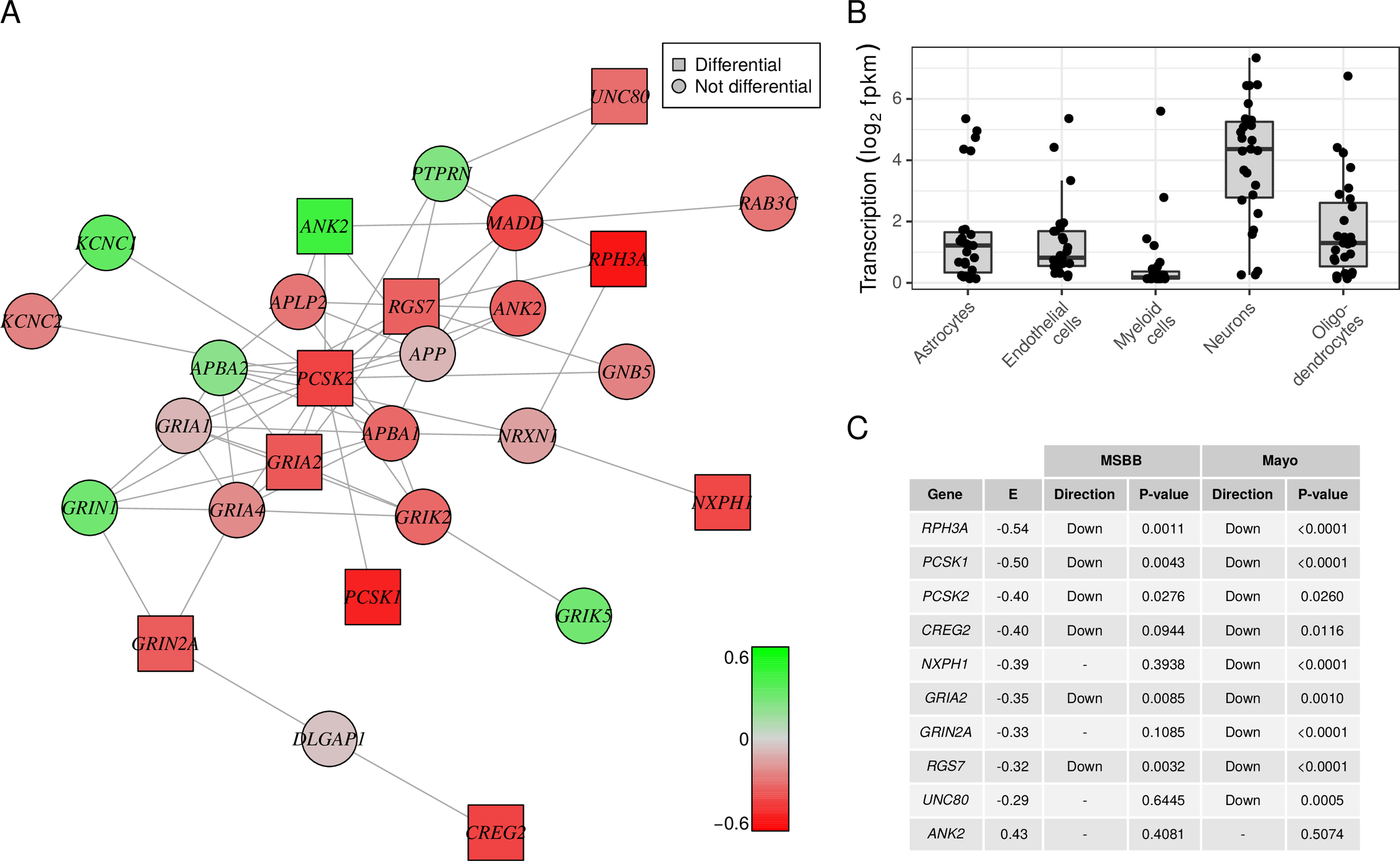

Supplement: S4 Fig — (A) Graph shows the subnetwork of differential genes largely involved in synaptic signaling. Color encodes the value of the integrative statistic from green (upregulated in AD) to red (downregulated in AD). Squares indicate significantly differential genes (99% credible interval). The gene ANK2 is represented twice reflecting two alternative active promoters. (B) Boxplots depict the transcription levels of the subnetwork’s genes in each of five major brain cell types obtained from an external RNA-seq dataset of purified cell types. (C) Table shows the value of the integrative statistic E^i and the unadjusted p-value from the two external validation datasets for each significant gene in the subnetwork. The directionality in the validation studies (up- or downregulated in AD) is given if the p-value was less than 0.1. (TIF) [file pcbi.1007771.s004.tif]
