# Supplementary material for: Bayesian integrative analysis of epigenomic and transcriptomic data identifies Alzheimer's disease candidate genes and networks
Source: PLoS Comput Biol. 2020 Apr 7;16(4):e1007771. doi: 10.1371/journal.pcbi.1007771 (PMC7138305; doi:10.1371/journal.pcbi.1007771)
Supplement: S1 Table — (DOCX) [file pcbi.1007771.s005.docx]

**S1 Table. Hyperparameters of the hierarchical Bayesian model.**

| Hyperparameter | Main analysis | Simulation |
| --- | --- | --- |
| $a_{\sigma}$ | $1.054\times{10}^{-5}$ | $1.103\times{10}^{-5}$ |
| $b_{\sigma}$ | $3.246\times{10}^{-5}$ | $3.322\times{10}^{-5}$ |
| $a_{\tilde{\nu}}$ | $1.639\times{10}^{-3}$ | $2.758\times{10}^{-4}$ |
| $b_{\tilde{\nu}}$ | $4.048\times{10}^{-4}$ | $1.661\times{10}^{-5}$ |
| $a_{\nu_{H}}$ | $1.127\times{10}^{-2}$ | $4.109\times{10}^{-3}$ |
| $b_{\nu_{H}}$ | $1.061\times{10}^{-3}$ | $6.411\times{10}^{-4}$ |

Table shows the hyperparameters calculated for the main analysis and for the simulation study using the empirical Bayes approach described in the methods.
