# Supplementary material for: Bayesian integrative analysis of epigenomic and transcriptomic data identifies Alzheimer's disease candidate genes and networks
Source: PLoS Comput Biol. 2020 Apr 7;16(4):e1007771. doi: 10.1371/journal.pcbi.1007771 (PMC7138305; doi:10.1371/journal.pcbi.1007771)
Supplement: S2 Table — (DOCX) [file pcbi.1007771.s006.docx]

**S2 Table. Parameter estimates of the hierarchical Bayesian model.**

| Variable | Estimated value | 99% credible interval |
| --- | --- | --- |
| $\beta_{0}$ | $-1.433\times{10}^{-2}$ | $[-2.070\times{10}^{-2}, -7.640\times{10}^{-3}]$ |
| $\nu_{H}$ | $4.405\times{10}^{-2}$ | $[ 4.134\times{10}^{-2}, 4.688\times{10}^{-2}]$ |
| $\tilde{\nu}$ | $1.349\times{10}^{-2}$ | $[8.610\times{10}^{-3}, 1.863\times{10}^{-2}]$ |

Table shows the median (2^nd^ column) and the 99% credible interval (3^rd^ column) from the posterior distributions of the model variables stated in the first column.
