# Supplementary material for: Bayesian integrative analysis of epigenomic and transcriptomic data identifies Alzheimer's disease candidate genes and networks
Source: PLoS Comput Biol. 2020 Apr 7;16(4):e1007771. doi: 10.1371/journal.pcbi.1007771 (PMC7138305; doi:10.1371/journal.pcbi.1007771)
Supplement: S4 Table — (DOCX) [file pcbi.1007771.s008.docx]

**S4 Table. GO analysis of the myeloid cell differentiation subnetwork.**

| GO ID | GO Term | # GO | # Net | # Exp. | P-Value |
| --- | --- | --- | --- | --- | --- |
| GO:0030099 | myeloid cell differentiation | 221 | 8 | 0.36 | 6.80 × 10^-10^ |
| GO:0030097 | hemopoiesis | 401 | 8 | 0.66 | 7.40 × 10^-8^ |
| GO:0048534 | hematopoietic or lymphoid organ development | 417 | 8 | 0.68 | 1.00 × 10^-7^ |
| GO:0007260 | tyrosine phosphorylation of STAT protein | 31 | 4 | 0.05 | 1.50 × 10^-7^ |
| GO:0002520 | immune system development | 443 | 8 | 0.73 | 1.60 × 10^-7^ |
| GO:0018108 | peptidyl-tyrosine phosphorylation | 192 | 6 | 0.32 | 3.70 × 10^-7^ |
| GO:0018212 | peptidyl-tyrosine modification | 195 | 6 | 0.32 | 4.00 × 10^-7^ |
| GO:1903706 | regulation of hemopoiesis | 207 | 6 | 0.34 | 5.70 × 10^-7^ |
| GO:0002521 | leukocyte differentiation | 218 | 6 | 0.36 | 7.80 × 10^-7^ |
| GO:1902105 | regulation of leukocyte differentiation | 115 | 5 | 0.19 | 8.30 × 10^-7^ |
| GO:0046425 | regulation of JAK-STAT cascade | 50 | 4 | 0.08 | 1.10 × 10^-6^ |
| GO:1904892 | regulation of STAT cascade | 52 | 4 | 0.09 | 1.30 × 10^-6^ |
| GO:0071310 | cellular response to organic substance | 1419 | 11 | 2.33 | 1.30 × 10^-6^ |
| GO:0031295 | T cell costimulation | 17 | 3 | 0.03 | 2.40 × 10^-6^ |
| GO:0007259 | JAK-STAT cascade | 64 | 4 | 0.11 | 2.90 × 10^-6^ |
| GO:0031294 | lymphocyte costimulation | 18 | 3 | 0.03 | 2.90 × 10^-6^ |
| GO:0002684 | positive regulation of immune system process | 447 | 7 | 0.73 | 3.30 × 10^-6^ |
| GO:0097696 | STAT cascade | 66 | 4 | 0.11 | 3.30 × 10^-6^ |
| GO:0001817 | regulation of cytokine production | 291 | 6 | 0.48 | 4.20 × 10^-6^ |
| GO:0030225 | macrophage differentiation | 22 | 3 | 0.04 | 5.50 × 10^-6^ |

The first two columns show GO IDs and the respective GO terms. The third column shows the total number of genes in our analysis that were associated with the GO term. Columns four and five show the observed and expected number of genes in the subnetwork that were associated with the GO term. The last column shows the unadjusted enrichment p-value. The top 20 GO terms sorted by p-value are shown.
