# Supplementary material for: Bayesian integrative analysis of epigenomic and transcriptomic data identifies Alzheimer's disease candidate genes and networks
Source: PLoS Comput Biol. 2020 Apr 7;16(4):e1007771. doi: 10.1371/journal.pcbi.1007771 (PMC7138305; doi:10.1371/journal.pcbi.1007771)
Supplement: S5 Table — (DOCX) [file pcbi.1007771.s009.docx]

**S5 Table. GO analysis of the protein phosphorylation network.**

| GO ID | GO Term | # GO | # Net | # Exp. | P-Value |
| --- | --- | --- | --- | --- | --- |
| GO:0006468 | protein phosphorylation | 1035 | 19 | 4.89 | 6.7 × 10^-8^ |
| GO:0018105 | peptidyl-serine phosphorylation | 172 | 8 | 0.81 | 1.2 × 10^-6^ |
| GO:0000226 | microtubule cytoskeleton organization | 313 | 10 | 1.48 | 1.5 × 10^-6^ |
| GO:0018209 | peptidyl-serine modification | 182 | 8 | 0.86 | 1.8 × 10^-6^ |
| GO:0035556 | intracellular signal transduction | 1557 | 21 | 7.35 | 2.0 × 10^-6^ |
| GO:0016310 | phosphorylation | 1290 | 19 | 6.09 | 2.2 × 10^-6^ |
| GO:0007010 | cytoskeleton organization | 778 | 14 | 3.67 | 8.1 × 10^-6^ |
| GO:0007017 | microtubule-based process | 435 | 10 | 2.05 | 2.7 × 10^-5^ |
| GO:0006796 | phosphate-containing compound metabolic process | 1865 | 21 | 8.8 | 3.7 × 10^-5^ |
| GO:0006793 | phosphorus metabolic process | 1884 | 21 | 8.89 | 4.3 × 10^-5^ |
| GO:0010822 | positive regulation of mitochondrion organization | 86 | 5 | 0.41 | 4.9 × 10^-5^ |
| GO:0032270 | positive regulation of cellular protein metabolic process | 880 | 13 | 4.15 | 1.5 × 10^-4^ |
| GO:0032436 | positive regulation of proteasomal ubiquitin-dependent protein catabolic process | 59 | 4 | 0.28 | 1.6 × 10^-4^ |
| GO:1901030 | positive regulation of mitochondrial outer membrane permeabilization involved in apoptotic signaling pathway | 25 | 3 | 0.12 | 2.1 × 10^-4^ |
| GO:1903827 | regulation of cellular protein localization | 356 | 8 | 1.68 | 2.3 × 10^-4^ |
| GO:0051247 | positive regulation of protein metabolic process | 932 | 13 | 4.4 | 2.6 × 10^-4^ |
| GO:2000060 | positive regulation of ubiquitin-dependent protein catabolic process | 67 | 4 | 0.32 | 2.7 × 10^-4^ |
| GO:0061136 | regulation of proteasomal protein catabolic process | 126 | 5 | 0.59 | 3.0 × 10^-4^ |
| GO:0023052 | signaling | 3219 | 27 | 15.19 | 3.0 × 10^-4^ |
| GO:0007154 | cell communication | 3242 | 27 | 15.3 | 3.4 × 10^-4^ |

The first two columns show GO IDs and the respective GO terms. The third column shows the total number of genes in our analysis that were associated with the GO term. Columns four and five show the observed and expected number of genes in the subnetwork that were associated with the GO term. The last column shows the unadjusted enrichment p-value. The top 20 GO terms sorted by p-value are shown.
