# Supplementary material for: Bayesian integrative analysis of epigenomic and transcriptomic data identifies Alzheimer's disease candidate genes and networks
Source: PLoS Comput Biol. 2020 Apr 7;16(4):e1007771. doi: 10.1371/journal.pcbi.1007771 (PMC7138305; doi:10.1371/journal.pcbi.1007771)
Supplement: S6 Table — (DOCX) [file pcbi.1007771.s010.docx]

**S6 Table. GO analysis of the synaptic signaling network.**

| GO ID | GO Term | # GO | # Net | # Exp. | P-Value |
| --- | --- | --- | --- | --- | --- |
| GO:0035235 | ionotropic glutamate receptor signaling pathway | 17 | 8 | 0.06 | 4.6 × 10^-16^ |
| GO:0060079 | excitatory postsynaptic potential | 60 | 9 | 0.23 | 7.4 × 10^-13^ |
| GO:0099565 | chemical synaptic transmission, postsynaptic | 65 | 9 | 0.25 | 1.6 × 10^-12^ |
| GO:0060078 | regulation of postsynaptic membrane potential | 81 | 9 | 0.31 | 1.2 × 10^-11^ |
| GO:0042391 | regulation of membrane potential | 242 | 12 | 0.92 | 4.5 × 10^-11^ |
| GO:0007215 | glutamate receptor signaling pathway | 63 | 8 | 0.24 | 6.4 × 10^-11^ |
| GO:0007267 | cell-cell signaling | 881 | 16 | 3.34 | 3.6 × 10^-8^ |
| GO:0007268 | chemical synaptic transmission | 438 | 12 | 1.66 | 3.9 × 10^-8^ |
| GO:0098916 | anterograde trans-synaptic signaling | 438 | 12 | 1.66 | 3.9 × 10^-8^ |
| GO:0099537 | trans-synaptic signaling | 442 | 12 | 1.68 | 4.3 × 10^-8^ |
| GO:0099536 | synaptic signaling | 446 | 12 | 1.69 | 4.8 × 10^-8^ |
| GO:0034220 | ion transmembrane transport | 574 | 13 | 2.18 | 8.6 × 10^-8^ |
| GO:0006811 | ion transport | 821 | 14 | 3.12 | 7.9 × 10^-7^ |
| GO:0050804 | modulation of chemical synaptic transmission | 291 | 9 | 1.1 | 9.9 × 10^-7^ |
| GO:0099177 | regulation of trans-synaptic signaling | 292 | 9 | 1.11 | 1.0 × 10^-6^ |
| GO:0014075 | response to amine | 25 | 4 | 0.09 | 2.1 × 10^-6^ |
| GO:0035249 | synaptic transmission, glutamatergic | 58 | 5 | 0.22 | 2.4 × 10^-6^ |
| GO:1905114 | cell surface receptor signaling pathway involved in cell-cell signaling | 327 | 9 | 1.24 | 2.6 × 10^-6^ |
| GO:0055085 | transmembrane transport | 773 | 13 | 2.93 | 2.6 × 10^-6^ |
| GO:1901018 | positive regulation of potassium ion transmembrane transporter activity | 14 | 3 | 0.05 | 1.8 × 10^-5^ |

The first two columns show GO IDs and the respective GO terms. The third column shows the total number of genes in our analysis that were associated with the GO term. Columns four and five show the observed and expected number of genes in the subnetwork that were associated with the GO term. The last column shows the unadjusted enrichment p-value. The top 20 GO terms sorted by p-value are shown.
