# Supplementary material for: Bayesian integrative analysis of epigenomic and transcriptomic data identifies Alzheimer's disease candidate genes and networks
Source: PLoS Comput Biol. 2020 Apr 7;16(4):e1007771. doi: 10.1371/journal.pcbi.1007771 (PMC7138305; doi:10.1371/journal.pcbi.1007771)
Supplement: S1 File — BUGS code for the hierarchical Bayesian model. (PDF) [file pcbi.1007771.s011.pdf]

S1 Supporting Information

ROS/MAP targeted proteomics dataset

The ROS/MAP targeted proteomics dataset was generated from dorsolateral prefrontal cortex specimens of 1,226 study participants and includes 186 peptides of 126 distinct genes measured in two batches. Protein targets were selected based on evidence from literature and other datasets suggesting an involvement in AD [1]. The dataset and a description of the data generation and normalization process is available at NIA's Accelerating Medicine Partnership – Alzheimer's Disease (AMP-AD) Knowledge Portal (Synapse ID: syn10468856). For this study, we selected samples that fulfilled our case/control definition described in the methods of the main article (n=393 AD, n=214 controls). Then, a linear regression model with the normalized peptide abundance as outcome and AD/control as independent variable was fitted for each peptide. Age, gender and postmortem interval were added to the model as additional covariates. If multiple peptides of the same protein were measured, we followed the suggestion from the original publication and selected the peptide with the largest signal to noise ratio [1]. To compare the results from the protein data to our integrative analysis, we extracted the coefficient of the AD variable from the regression model and plotted the value against the integrative statistic $\hat{E}_i$ of the same gene (Fig 3A). If a gene had multiple active promoters and therefore mapped to multiple features in our integrative analysis, we averaged the respective $\hat{E}_i$ values. In total, 98 distinct genes were included in the protein data and in the integrative analysis.

Mount Sinai Brain Bank (MSBB) study transcription dataset

The MSBB dataset [2] was used to remove edges from the functional gene similarity network which are unlikely to be valid in the human brain as described in the Methods of the main manuscript. For pruning the network, we did not restrict the dataset to AD and control samples, but also included subjects with other diagnosis such as mild cognitive impairment. Further, we used all four brain regions (Brodmann areas 10, 22, 36 and 44) profiled by the MSBB study to calculate pairwise correlations between genes. To minimize the effect of technical variables, pairwise correlations were calculated on residuals obtained from regressing transcription values on technical variables, age and gender. The residual dataset is available at Synapse (Synapse ID: syn8485027). Details about outlier removal, normalization and calculation of the residuals are described elsewhere [3].

The MSBB dataset was also used to validate differential genes identified by our study. For validation, only samples from the inferior frontal gyrus (Brodmann area 44) were included. AD and control cases were defined based on Clinical Dementia Rating (CDR), CERAD score and Braak stage. AD cases (n=79) were defined as patients with a CDR of *mild*, *moderate*, *severe*, *profound*, or *terminal dementia*, a CERAD score indicating *possible*, *probable* or *definite AD*, and a Braak stage ≥ 4. Controls (n=37) were defined as samples with a CDR of *no* or *questionable dementia*, a *normal* CERAD score, and a Braak stage ≤ 3. At the RNA-seq data preprocessing and normalization step, genes with less than 1 CPM (read Counts Per Million total reads) in at least 50% of the AD samples and in at least 50% of the control samples were removed. After filtering, 747 of our differential genes from the integrative analysis of the DLPFC

ROS/MAP data were included in the MSBB data. Data normalization and analysis of the MSBB data were conducted by the AMP-AD consortium. Details and analysis results are publically available at the AMP-AD Knowledge Portal (Synapse ID: syn14237651).

Mayo LOAD study transcription dataset

To validate our results in the Mayo LOAD dataset [4], only samples from the temporal cortex were selected. AD cases (n=80) met the NINCDS-ADRDA criteria of *definite AD* and demonstrated a Braak stage $\geq$ 4. Controls (n=71) were pathologically characterized by a Braak stage $\leq$ 3, a CERAD score indicating *no* or *sparse neuritic plaques*, and had none of the following pathologic diagnoses: Alzheimer's disease, Parkinson's disease, Dementia with Lewy bodies, vascular dementia, progressive supranuclear palsy, motor neuron disease, corticobasal degeneration, Pick's disease, Huntington's disease, frontotemporal lobar degeneration, hippocampal sclerosis or dementia lacking distinctive histology. At the RNA-seq data preprocessing and normalization step, genes with less than 1 CPM (read Counts Per Million total reads) in at least 50% of the AD samples and in at least 50% of the control samples were removed. After filtering, 759 of our differential genes from the integrative analysis of the DLPFC ROS/MAP data were included in the Mayo LOAD data. Data normalization and analysis of the Mayo LOAD data were conducted by the AMP-AD consortium. Details and analysis results are publically available at the AMP-AD Knowledge Portal (Synapse ID: syn14237651).

The Mayo LOAD dataset was also used to study the three differential networks identified in the integrative analysis. For this analysis, we included n=30 individuals diagnosed with pathologic aging in addition to the n=80 AD and n=71 control cases. Individuals with pathologic aging lacked the same pathologic diagnoses as controls (described above) and had a Braak stage $\leq$ 3, but had a CERAD score indicating *moderate* or *frequent neuritic plaques*. None of the individuals with pathologic aging had a clinical diagnosis of dementia or mild cognitive impairment. To summarize the transcription level of a network, we centered the normalized transcription levels of the network's genes and then calculated the first principal component of the sample × gene matrix. If the first principal component was negatively correlated with the vector of mean transcription levels of the network, the principal component was multiplied by -1 such that a large value corresponded to a large overall transcription level of the network.

ROS/MAP single-nucleus transcription data

The ROS/MAP single-nucleus RNA-sequencing (snRNA-seq) data were generated from dorsolateral prefrontal cortex specimens of n=48 study participants and contained on average 1,680 nuclei per individual [5]. The dataset and a description of the data generation and normalization is available at NIA's Accelerating Medicine Partnership – Alzheimer's Disease (AMP-AD) Knowledge Portal (Synapse ID: syn18485175). Cell types were clustered and annotated as described in the original publication [5]. Cell-type-specific transcription values for astrocytes, excitatory neurons, inhibitory neurons, microglia, oligodendrocytes and oligodendrocyte progenitor cells were calculated as follows: For each individual and gene, we added the RNA-seq reads from all cells of the same cell type. Raw counts were normalized for sequencing depth using the TMM method [6]. Subsequently, normalized counts per million (cpm)

were log_2-transformed. All n=48 samples were used in Fig 5D and Fig 5E, whereas only a subset of n=29 samples that fulfilled either our definition for AD or control samples described in the methods section of the main manuscript was used in Fig 5F.

Data acknowledgement statements

Mount Sinai Brain Bank (MSBB) study. Study data were generated from postmortem brain tissue collected through the Mount Sinai VA Medical Center Brain Bank and were provided by Dr. Eric Schadt from Mount Sinai School of Medicine.

Mayo LOAD study. Study data were provided by the following sources: The Mayo Clinic Alzheimer's Disease Genetic Studies, led by Dr. Nilufer Taner and Dr. Steven G. Younkin, Mayo Clinic, Jacksonville, FL using samples from the Mayo Clinic Study of Aging, the Mayo Clinic Alzheimer's Disease Research Center, and the Mayo Clinic Brain Bank. Data collection was supported through funding by NIA grants P50 AG016574, R01 AG032990, U01 AG046139, R01 AG018023, U01 AG006576, U01 AG006786, R01 AG025711, R01 AG017216, R01 AG003949, NINDS grant R01 NS080820, CurePSP Foundation, and support from Mayo Foundation. Study data includes samples collected through the Sun Health Research Institute Brain and Body Donation Program of Sun City, Arizona. The Brain and Body Donation Program is supported by the National Institute of Neurological Disorders and Stroke (U24 NS072026 National Brain and Tissue Resource for Parkinson's Disease and Related Disorders), the National Institute on Aging (P30 AG19610 Arizona Alzheimer's Disease Core Center), the Arizona Department of Health Services (contract 211002, Arizona Alzheimer's Research Center), the Arizona Biomedical Research Commission (contracts 4001, 0011, 05-901 and 1001 to the Arizona Parkinson's Disease Consortium) and the Michael J. Fox Foundation for Parkinson's Research.

Implementation of the hierarchical Bayesian model in BUGS

```
model{

   for(i in 1:N){                          # genes
      for(j in 1:M){                       # individuals
         Z[i, j] ~ dnorm(mu[i], tau[i])
      }
      mu[i] <- beta0 + U[i] + H[i]
      tau[i] ~ dgamma(tau.a, tau.b)
      H[i] ~ dnorm(0, tau.h)
   }

   U[1:N] ~ car.normal(adj[], weights[], num[], tau.u)
   tau.u ~ dgamma(tau.u.a, tau.u.b)
   tau.h ~ dgamma(tau.h.a, tau.h.b)
   beta0 ~ dflat()

}
```

References

1. Yu L, Petyuk VA, Gaiteri C, Mostafavi S, Young-Pearse T, Shah RC, et al. Targeted brain proteomics uncover multiple pathways to Alzheimer's dementia. Ann Neurol. 2018;84(1):78-88. Epub 2018/06/17. doi: 10.1002/ana.25266. PubMed PMID: 29908079; PubMed Central PMCID: PMCPMC6119500.
2. Wang M, Beckmann ND, Roussos P, Wang E, Zhou X, Wang Q, et al. The Mount Sinai cohort of large-scale genomic, transcriptomic and proteomic data in Alzheimer's disease. Sci Data. 2018;5:180185. Epub 2018/09/12. doi: 10.1038/sdata.2018.185. PubMed PMID: 30204156; PubMed Central PMCID: PMCPMC6132187.
3. Logsdon B, Perumal TM, Swarup V, Wang M, Funk C, Gaiteri C, et al. Meta-analysis of the human brain transcriptome identifies heterogeneity across human AD coexpression modules robust to sample collection and methodological approach. bioRxiv. 2019.
4. Allen M, Carrasquillo MM, Funk C, Heavner BD, Zou F, Younkin CS, et al. Human whole genome genotype and transcriptome data for Alzheimer's and other neurodegenerative diseases. Sci Data. 2016;3:160089. Epub 2016/10/12. doi: 10.1038/sdata.2016.89. PubMed PMID: 27727239; PubMed Central PMCID: PMCPMC5058336.
5. Mathys H, Davila-Velderrain J, Peng Z, Gao F, Mohammadi S, Young JZ, et al. Single-cell transcriptomic analysis of Alzheimer's disease. Nature. 2019;570(7761):332-7. Epub 2019/05/03. doi: 10.1038/s41586-019-1195-2. PubMed PMID: 31042697; PubMed Central PMCID: PMCPMC6865822.
6. Robinson MD, Oshlack A. A scaling normalization method for differential expression analysis of RNA-seq data. Genome Biol. 2010;11(3):R25. Epub 2010/03/04. doi: 10.1186/gb-2010-11-3-r25. PubMed PMID: 20196867; PubMed Central PMCID: PMCPMC2864565.
